# Initiation of mRNA translation in bacteria: structural and dynamic aspects

**DOI:** 10.1007/s00018-015-2010-3

**Published:** 2015-08-11

**Authors:** Claudio O. Gualerzi, Cynthia L. Pon

**Affiliations:** Laboratory of Genetics, University of Camerino, 62032 Camerino, Italy

**Keywords:** Protein synthesis, Translation initiation factors, mRNA initiation region, fMet-tRNA, GTP

## Abstract

Initiation of mRNA translation is a major checkpoint for regulating level and fidelity of protein synthesis. Being rate limiting in protein synthesis, translation initiation also represents the target of many post-transcriptional mechanisms regulating gene expression. The process begins with the formation of an unstable 30S pre-initiation complex (30S pre-*IC*) containing initiation factors (IFs) IF1, IF2 and IF3, the translation initiation region of an mRNA and initiator fMet-tRNA whose codon and anticodon pair in the P-site following a first-order rearrangement of the 30S pre-*IC* produces a locked 30S initiation complex (30S*IC*); this is docked by the 50S subunit to form a 70S complex that, following several conformational changes, positional readjustments of its ligands and ejection of the IFs, becomes a 70S initiation complex productive in initiation dipeptide formation. The first EF-G-dependent translocation marks the beginning of the elongation phase of translation. Here, we review structural, mechanistic and dynamical aspects of this process.

## Introduction

Initiation of mRNA translation is normally the rate-limiting step of protein synthesis in bacteria and, as such, represents the target of the post-transcriptional regulation of expression of a large number of genes [1–4]; it also plays a significant role in determining mRNA stability [5, 6].

The initiation phase of translation begins with the formation of a 30S initiation complex (30S*IC*) in which the start codon of the mRNA translation initiation region (TIR) is decoded by the CAU anticodon of the initiator fMet-tRNA in the P-site of the small (30S) ribosomal subunit. The 30S*IC* is then joined by the large (50S) ribosomal subunit to yield a 70S initiation complex (70S*IC*) capable of forming an “initiation dipeptide” with the aminoacyl-tRNA encoded by the second mRNA codon carried to the ribosomal A-site by elongation factor EF-Tu. Three proteins, the initiation factors (IFs) IF1, IF2, and IF3, determine the kinetics and fidelity of the overall initiation process. The three IFs are bound, one copy each, to specific sites of the 30S subunit and after assisting 30S*IC* formation are dissociated from the ribosome during the transition 30S*IC* → 70S*IC* (see below). IF2 is the last factor to be dissociated, leaving the ribosome after having positioned fMet-tRNA in the P-site of a 70S*IC* so as to be productive as a donor in initiation dipeptide formation. The first EF-G-dependent translocation of the initiation dipeptide marks the beginning of the elongation phase of protein synthesis (for previous reviews on the subject see [7–9]).

## The actors on the translation initiation stage

In bacteria, the initiation phase of protein synthesis involves a limited number of “actors”. Aside from the two ribosomal subunits, key roles are played by the initiator tRNA_fmet_, the TIR of the mRNA and three protein factors, the initiation factors (IFs) IF1, IF2 and IF3 that ensure speed and accuracy to the overall process [7–9]. The initiator tRNA_fmet_ participates in the process after having been aminoacylated with methionine and formylated, two enzymatic reactions that yield fMet-tRNA. Since the tRNA synthetase that aminoacylates initiator tRNA_fmet_ is the same that aminoacylates elongator tRNA_met_, the only additional protein having a direct bearing on initiation is transformylase that uses Met-tRNA_fmet_ as a specific substrate to transfer a formyl group to the αNH_2_ of methionine [10].

The bacterial cell produces and expresses a plethora of different mRNAs with different TIR sequences and structures; the efficiency by which these individual transcripts are translated depends not only upon their abundance and stability but also upon the nature of their translation initiation region (TIR). Thus, unlike the other aforementioned actors that represent constants, the mRNA TIRs represent essentially the only variable in the process of mRNA initiation site selection.

## Properties of the mRNA translation initiation regions (TIRs)

Although the triplet AUG is by far the most frequent initiation codon found in TIRs, other initiation triplets (i.e., GUG, UUG, AUU, AUC, and AUA) are found in bacteria and the central U is the only universally conserved base of the start codon. Among the aforementioned triplets, those having a 3′-G (i.e., AUG, GUG and UUG) are recognized as “canonical” insofar as they are not subject to discrimination by IF3 unlike the other “non-canonical” triplets [11]. The AUG initiation codon is important, not only for being decoded by initiator tRNA in 5′-leadered mRNAs, but also to serve as a strong signal to allow translation of leaderless (see below) mRNAs. None of the other potential start codons (i.e., GUG, UUG or CUG) can substitute AUG in this function for which the AUG triplet is important “per se” and not because of its complementarity to the initiator tRNA anticodon. In fact, codon–anticodon pairing at the 5′end of the leaderless mRNA is not sufficient to elicit translation because an initiator tRNA with compensating anticodon mutations was unable to restore the expression of leaderless mRNA bearing a UAG start codon [12].

Another important characteristic of a large number of bacterial mRNA TIRs is the presence of the Shine–Dalgarno (or SD) sequence complementary to the 3′ end sequence of 16S rRNA (the anti-SD sequence or aSD). To ensure efficient translation, this sequence must be present at an optimal distance (i.e., 4–9 nucleotides in *Escherichia coli*) upstream of the initiation codon [13] although this distance can also be quite longer.

**Fig. 1:**
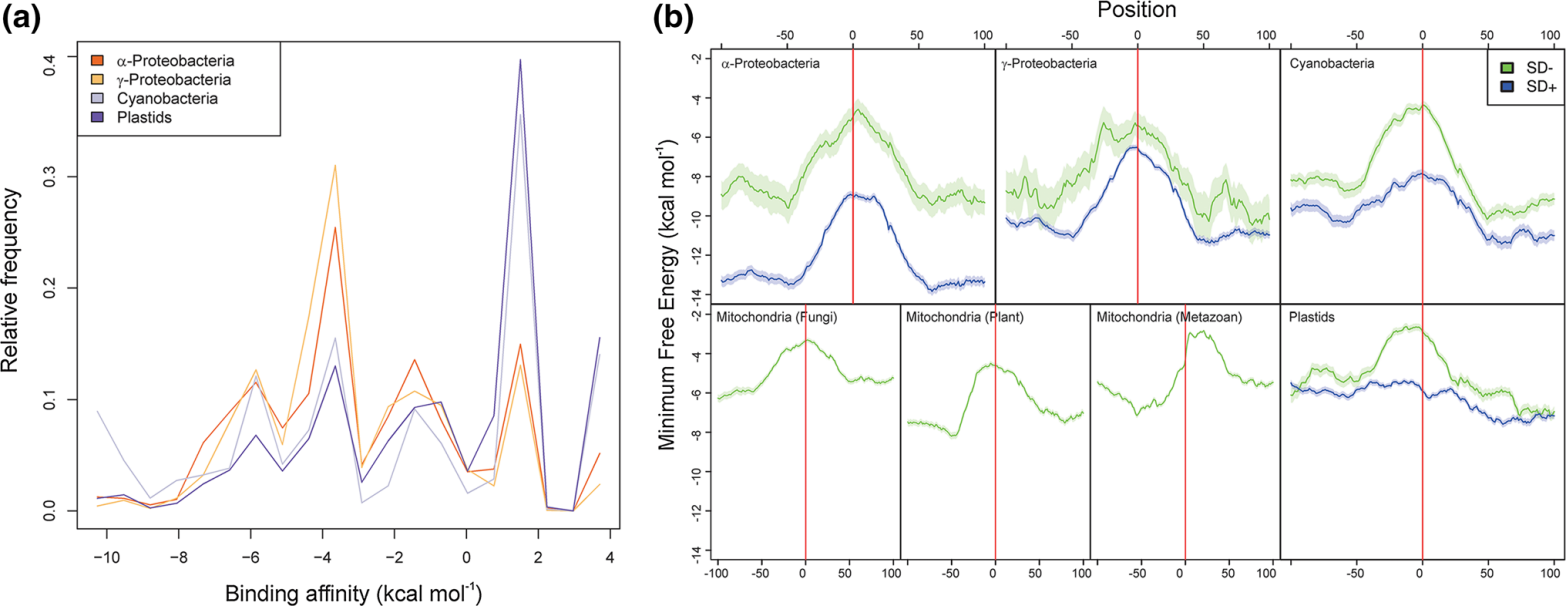
Distribution of SD-containing and SD-lacking mRNAs in the bacterial kingdom and deficit of RNA secondary structure near the start codon. **a** Normalized distributions of energies assessed for hybridizations between the anti-SD of 16S rRNAs and the −22 to −2 sequences of 5′ UTRs of α-proteobacterial, γ-proteo-bacterical, cyanobacterial and plastid genes. Four major peaks at −5.9, −3.6, −1.4 and +1.5 kcal mol^−1^ are visible in all taxonomic groups. They correspond, from *left* to *right*, to: (1) mRNAs with SD sequence AGGAG, (2) mRNAs with SD sequence GAGG, AGGA or GGAG, (3) mRNAs with short SD-like sequences (AGG, GAG or GGA), which may engage in SD-type interactions with the 3′ end of 16S rRNA and (4) mRNAs without SD sequences. **b** Predicted amount of RNA secondary structure around the start codon in α-proteobacteria, γ-proteobacteria, cyanobacteria, plant, metazoan and fungal mitochondria, and plastids. The line shows the running mean Minimum Free Energy (standard error of the mean is indicated by the shaded area) of 5000 genes with (*blue*) and without (*green*) an SD sequence, the difference in their MFE upstream and downstream of the initiation region (0 = first start codon nucleotide) in the three bacterial groups being largely due to differences in AT-content between genomes. In metazoan mitochondria, most transcripts are leaderless and lack a 5′ UTR so that the minimum free energy peak is shifted into the coding region. Reproduced with permission from [18]

The role played by the SD sequence as the most important element governing various aspects of translation initiation (efficiency, reading frame selection, regulation) was taken as dogma, often based on circumstantial evidence. On the other hand, data and considerations casting doubts on the actual extent of its relevance were often ignored; it appeared clear from the very beginning that the existence of mRNAs completely lacking the SD sequence indicated that this sequence is neither necessary nor sufficient for translation initiation [14, 15]. Furthermore, whereas “SD sequences” could be as frequent as Gly (GGA and GGU) or Arg (AGG) codons, the mere presence of an SD or of an SD-like sequence followed by an AUG triplet does not ensure translation initiation [14]. Nevertheless, that the SD–aSD pairings play a role in initiating translation of a large number of mRNAs is now established beyond any possible doubt. However, the circumstance that both role and importance of the SD sequence were deduced primarily from studies carried out with *E. coli* may have contributed to generate a biased impression concerning the relevance of the SD sequence. Indeed, if one considers the entire bacterial kingdom, it is clear that SD sequences are not ubiquitous and that only a minor fraction of bacterial mRNAs contain an SD sequence. For instance, the entire Gram-negative bacterial phylum Bacteroidetes does not use SD interactions to initiate translation [16]. Leaderless mRNAs and “leadered” mRNAs lacking an SD sequence are at least each as common as SD-containing mRNAs [17], a conclusion confirmed by a recent genome-wide search for SD-independent translation in bacterial and organellar genomes that revealed that a large fraction (15–100 %) of prokaryotic transcripts is translated by an SD-independent mechanism, either because the mRNAs have no 5′ UTR (leaderless mRNAs) or because the 5′ UTR does not contain any SD-like sequence (Fig. 1a) [18].

Almost 15 years after the SD sequence was detected, the actual in vivo occurrence of SD–aSD base pairing was demonstrated through the use of “specialized ribosomes” bearing an SD sequence at the 3′ end of 16S rRNA and an aSD sequence in the TIR [19, 20]. The available data suggest that the SD–aSD duplex formed upstream the initiation triplet confers upon a given transcript an increased chance to outcompete other mRNAs for ribosomal binding [21] and offers an excellent way to increase the concentration of a potential start codon near the subunit’s P-site and enhance the thermodynamic affinity of a potentially productive 30S–mRNA complex [22]. However, successful and correct initiation site selection ultimately depends upon the kinetic parameters that govern the formation of the 30S*IC* and, subsequently, of the 70S*IC* [22, 23]. These parameters depend upon the overall nature of the TIR, the stability of its folding being of capital importance in determining translational efficiency [24]. Indeed, most mRNAs are highly structured and their coding sequences not accessible in a single-stranded form. Thus, even in the absence of an SD sequence, an AUG codon in an unstructured region of the mRNA (Fig. 1b) can unambiguously define the correct translation initiation site [25] also in light of the fact that the mechanistics of 30S initiation complex formation are not affected by the SD sequence whose presence is also not responsible for mRNA reading frame selection [22].

The extent to which the SD sequence determines translational efficiency of a given mRNA is a controversial issue. Genomic analysis suggested that highly expressed prokaryotic mRNAs are more likely to possess an SD sequence [26]. However, other data indicate that the importance of the SD could have been highly overestimated. For instance, an early finding showed that a long SD sequence (UAAGGAGG) is about four times more efficient in translation initiation ternary complex formation than a shorter (AAGGA) sequence [13]. On the other hand, more recent studies show that a too long SD sequence inhibits translation [27] and is discriminated against by IF3 [23]. Furthermore, the same SD sequence mutation reported to reduce by ~90 % bacteriophage T7 0.3 gene synthesis [28] did not cause more than 20–40 % reduction of the translational activity in vitro [29]. More recently, it was found that *lacZ* translation was reduced 15-fold upon changing the SD sequence from 5′-AGGA-3′ to 5′-UUUU-3′ but only two-fold after removal of the entire 5′ UTR [12]. A quantitatively similar reduction of protein synthesis level (i.e., no more than two-fold) was detected in vivo in a more recent study using “specialized” ribosomes [30], much less than originally estimated (>90 %) [19, 20]. It is possible that in vivo translation-independent mRNA decay caused by the lack of SD–aSD interaction [31–33] may have led to this overestimation.

In addition to its role in translation initiation, other functions have been attributed to the SD sequence aside from the aforementioned influence on transcript stability. For instance, an important role was attributed to internal SD sequences in allowing both −1 and +1 programmed ribosome frameshifting, the SD position with respect to the frameshifting site being different in the two cases [34]. Furthermore, SD-like features within mRNA coding sequences hybridizing with 16S rRNA of the translating ribosome were found to cause translational pausing and represent a major determinant of translation elongation rates. This is the likely reason why codons and codon pairs resembling canonical SD sites (see above) are disfavored in protein-coding sequences [35].

As mentioned above, a large number of bacterial genes, including genes that are expressed at high level, do not have a 5′ UTR or have just a few bases upstream the coding sequence that begins with a 5′ AUG [36]. Translation of these leaderless mRNAs likely involves an ancestral mechanism, conserved in bacteria, archaea and eukaryotic cells [36, 37] and depends on IF2 [36, 38], on 5′ phosphorylated AUG [12, 39, 40] and is antagonized by IF3 [41]. It has also been suggested that translation of these mRNAs begins with 70S monomers instead of 30S subunits [42–44].

Aside from the features of the mRNA TIRs so far described, the possible existence of several types of *cis*-acting elements functioning as translational enhancers, especially when no or weak SD sequences are present, has been sporadically reported (for an early review, see [2]). Among these *cis*-elements are the AU-rich stretches serving as recognition/binding sites for ribosomal protein S1 [33, 45]. Elements present on the 3′ side of the initiation codon among which the so-called downstream region (DR) [46, 47] and the downstream box (DB) [48] were also shown to affect translational efficiency. The hypothesis that the DB base pairs with 16S rRNA was shown to be inconsistent with empirical data. In fact, inversion of the 16S rRNA sequence suggested to base pair with the DB demonstrated that the 16S rRNA does not hybridize with its suggested target [49, 50].

The importance of base bias after the initiation codon in determining translational efficiency was examined in a number of in vitro and in vivo studies. For instance, the +2 codon immediately following the initiation codon was shown to increase the translational efficiency of an mRNA having a weak UUG start codon [46]. In the *E. coli* dihydrofolate reductase gene, the AAA and AAU triplets, occurring most frequently as second codons, were found to enhance translation efficiency, unlike codons occurring with lower frequency. Like in the case of the DB sequence, the effect of these “enhancer triplets” could not be attributed to mRNA–16S rRNA base-pairing [51]. Furthermore, several *E. coli* genes contain CA-rich sequences downstream the initiation triplet and stimulation of gene expression was observed when multimers of the CA dinucleotide were placed on the 3′ side of the start codon of several mRNAs, with and without 5′ UTR. The extent of the stimulation was a function of the number of the CA repeats introduced [52]. However, because the downstream CA multimers increase the mRNA affinity for the ribosome and the amount of full-length mRNA in vivo, it is likely that their effect is due not only to improved translational efficiency but also to an increased stability of the transcripts [52], as is the case for the AU-rich sequences within the 5′ UTR [33].

In conclusion, the numerous attempts to identify sequence and/or structural elements of mRNA that would determine its translational efficiency, be it with or without 5′ UTR, with or without SD sequence following various types of TIR mutations have led to a large number of conflicting results concerning the features that determine translational efficiency of mRNAs. Whereas there is no reason to doubt the validity of the conclusions reached in these studies, the large number of variables concerning primary, secondary and tertiary structures of the countless mRNA TIRs that may influence translation initiation prevents simple generalizations to be made. Thus, although it seems safe to state that the presence of a minimum level of secondary structure around the most common AUG start codon (Fig. 1b) and—whenever appropriate—the presence of medium-length SD sequence 5–7 bases upstream the start codon are conditions that favor translation initiation, predicting the translational effectiveness of a given RNA transcript remains a very uncertain task. The construction of systems for efficient synthesis of proteins, therefore, relies on empirical as much as theoretical considerations.

## The SD–aSD duplex: ribosomal localization, dissociation and mRNA shift

Several structural studies have dealt with the ribosomal localization of the SD–aSD duplex [53–56]. An early crystallographic study (at 7 Å resolution) localized an mRNA of 30 nucleotides in a groove encircling the small subunit neck showing the SD duplex accommodated between the subunit head and the back of the platform, in a large cleft constituted by elements of the 16S rRNA and of ribosomal proteins S11 and S18. In particular, at the bottom and to its left and right, the helix is surrounded by h20, by the 723 bulge loop and by h28 and h37; the major groove of the SD–aSD duplex contacts the basic and aromatic residues of the S18 NTD, while the NTD loop (Arg54) and the C-terminal tail of S11 contribute to forming the cleft with the latter contacting bases −4 to −6 of the mRNA [53]. In agreement with these data, subsequent crystallographic studies detected the SD duplex in a “chamber” between the subunit head and platform, in a position suitable for placing the AUG start codon in the immediate vicinity of the mRNA channel [55]; the duplex was seen to contact primarily h23a, h26, and h28 of 16S rRNA with the bulged U723 (h23a) interacting with the minor groove of the SD helix in correspondence with the C1539·G-10 base pair and the backbone of the “16S rRNA strand” of the duplex (nucleotides 1536–1539) contacting the basic N-terminal tail of S18. The presence of the SD–aSD duplex near the hinge of the subunit neck (helix 28) suggests that its formation may affect the position of the 30S subunit head, possibly reducing the mobility of platform and head and fixing the orientation of the latter so as to favor the optimal interaction of fMet-tRNA with the 30S P-site [56].

Comparison of the X-ray structures of ribosomal complexes corresponding to various phases of protein synthesis showed that an SD helix is still present after completion of the translation initiation step, and that it undergoes a clockwise 70° rotation accompanied by movement of the mRNA in the 3′ → 5′ direction and by a simultaneous lengthening of the SD duplex that now contacts ribosomal protein S2 [54, 56]. In both initiation and post-initiation complexes, the SD–aSD duplex anchors the mRNA 5′-end to the 30S platform whereas during elongation, the 5′ end of the mRNA becomes flexible after the dissociation of the SD–aSD duplex [54, 56].

The structures described above were obtained with crystals of *Thermus thermophilus* ribosomes that do not contain protein S21. Therefore, in *E. coli* ribosomes the SD–aSD helix cannot occupy the same position because it would sterically clash with protein S21 unless this protein occupies a different position in the presence of the SD duplex [54].

**Fig. 2:**
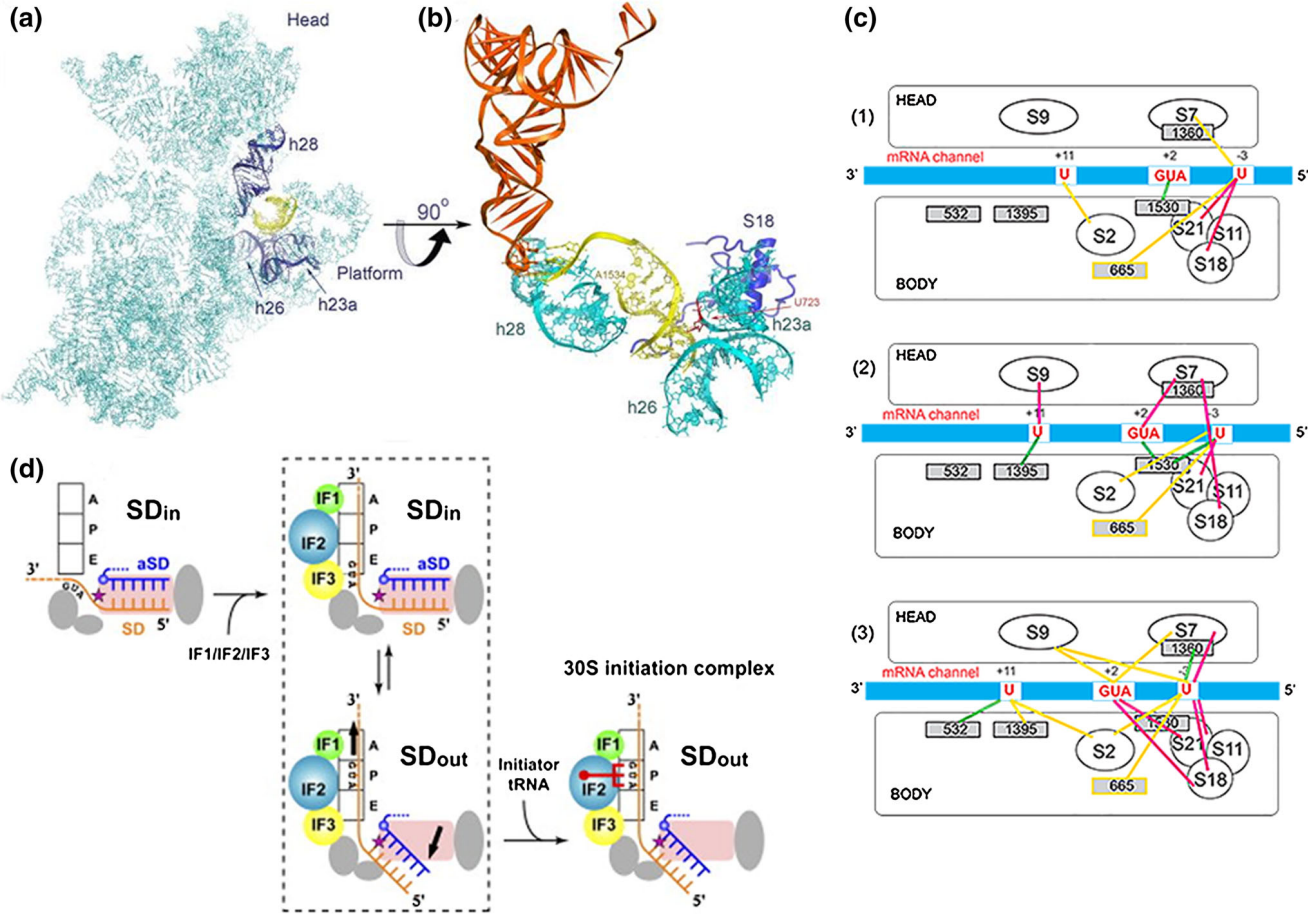
SD helix and mRNA movements on the 30S ribosomal subunit during translation initiation. **a** Location of the SD–aSD duplex (*yellow*) with respect to the 16S rRNA (*light blue*) within a 70S*IC*. The SD helix contacts h23a, h26 and h28 (*dark blue*); **b** close-up of the interaction between the SD helix (*yellow*) and h23a, h26 and h28 (*cyan*) and ribosomal protein S18 (*dark blue*). The bulged U723 that interacts with minor groove of the C1539·G10 bp and A1534 that binds to h28 in the 30S neck are indicated. The position of P-site-bound tRNA (*orange*) is also shown (reproduced with permission from [52]). Initiation factors-dependent and fMet-tRNA-dependent mRNA shift from “stand-by” to “P-decoding” site on the 30S subunit as evidenced by **c** site-directed cross-linking (redrawn from [60]) and **d** X-ray crystallography (reproduced from [55] with permission from Elsevier)

A relevant question concerns the timing of the SD–aSD dissociation. A sophisticated study in which the rupture force between a single ribosome complex and mRNA was measured by an optical tweezer assay led to the conclusion that the SD–aSD interaction is destabilized after formation of the first peptide bond and the grip of the ribosome on the mRNA is loosened [57]. However, these results should not be interpreted to mean that the SD–aSD interaction is dissociated at this stage. In fact, crystallography data indicate that the SD–aSD duplex is still intact after several codons have been translated [50]. In another study, the SD–aSD helix was reported to move along its screw axis during the first translocation step (Fig. 2a, b) [56]. Consistent with these data, the kinetics of initiation dipeptide formation were found to be hardly influenced by the presence/absence and length of the SD sequence whereas tripeptide formation proved substantially faster with an mRNA lacking the SD sequence compared to mRNAs with extended SD sequences (Rodnina M & Gualerzi CO, unpublished observation). These findings are compatible with the notion that after initiation dipeptide formation a strong SD–aSD interaction would slow down the first translocation step required for tripeptide formation. In addition, it should be recalled that the aSD sequence is accessible not only in the 30S subunits, but also in 70S monomers [58] and that elongation pauses whenever a translating ribosome encounters internal SD-like sequences in the mRNA [53].

Taken together, the above results indicate that the initial phase of protein synthesis is characterized by a dynamic interaction between the mRNA and the ribosome. A striking aspect of this dynamic behavior is represented by the IFs-induced mRNA shift originally demonstrated by binding competition between an SD octanucleotide and natural or synthetic mRNAs carried out in the presence of various combinations of IFs. These experiments demonstrated that the IFs affect only very marginally the thermodynamic stability of the 30S–mRNA complexes and do not influence the SD–aSD interaction, but influence instead the position of the mRNAs on the 30S subunit. The results suggested that in the absence of IFs the mRNA occupies a ribosomal “stand-by” site, likely comprising the region of the SD–aSD duplex, whereas in their presence the mRNA is shifted towards another ribosomal site with similar affinity for the mRNA, probably closer to the P-decoding site. Depending on the nature of the mRNA, this shift was mediated by IF2 and/or IF3, and favored by fMet-tRNA whose presence was not required [59]. Subsequently, the specific sites of mRNA, rRNA and ribosomal proteins interested by this shift were identified by site-directed crosslinking experiments. In particular, under the influence of IF3, the second position of the mRNA start codon and G1530 of 16S rRNA come in close proximity providing direct evidence for the occurrence of this IFs-induced partial relocation of the mRNA from the “stand-by” to the “decoding” site of the 30S subunit (Fig. 2c) [60]. These conclusions were fully confirmed by more recent crystallographic studies in which the mRNA was mapped in *T. thermophilus* ribosomal complexes corresponding to initiation, post-initiation and elongation phases of translation states (Fig. 2d) [54, 55]. Overall, it seems reasonable to hypothesize that the IFs-promoted movement of the mRNA on the 30S subunit favors the correct P-site decoding of the initiation triplet.

Aside from the movements of the SD duplex and the IFs-induced shift from stand-by to decoding site, other movements affecting the position of the mRNA occur on the ribosomal surface. Indeed, many mRNAs have elaborate structures at their 5′ UTR that may need to be unfolded and re-adjusted on the ribosomal subunit to expose the SD sequence (if present) and the start codon to the P-site, so as to become amenable for translation.

Overall, the mRNA–30S subunit interactions may be envisaged as a number of successive steps [61, 62]. If base pairings within a structured TIR are not too strong, the ribosome wins the competition with the mRNA structure and binds the template in a “stand-by site”, making use of the SD sequence, if this is present and properly exposed. The mRNA is then adjusted in the mRNA channel to allow P site decoding of the initiation triplet by fMet-tRNA. Alternatively, the mRNA binds to the ribosome in a “stand-by site”, making use of single-stranded regions transiently present in its 5′ UTR, possibly the AU-rich sequences that interact with ribosomal protein S1 [45]. According to at least one report [63], S1 is strategically located at the junction of head, platform, and main body of the 30S subunit on the “solvent side” of the subunit so as to capture mRNA nucleotides immediately upstream of the SD sequence. Subsequently, the SD sequence, if not already base-paired, is exposed stepwise by the S1 RNA unwinding activity [64] so as to base pair with the aSD sequence. The mRNA shift from stand-by to decoding site and the adjustment of the initiation codon in the P site that favors codon–anticodon interaction with fMet-tRNA stabilize the 30S initiation complex.

## The initiator tRNA

Bacterial tRNA_fMet_ is endowed with distinctive properties that distinguish it from the bulk elongator tRNAs and ensure its special role in translation initiation.

The initiator tRNA is first aminoacylated with methionine whose α-NH_2_ group is eventually blocked by a specific formyl transferase (TMF) to produce an fMet-tRNA molecule. This modification prevents interaction with elongation factor EF-Tu and ensures instead the recognition and binding of fMet-tRNA by initiation factor IF2. Furthermore, fMet-tRNA binds with high affinity to the ribosomal P-site, unlike all other aminoacyl-tRNAs that bind to the A-site in a ternary complex with EF-Tu and GTP. In the P-site, the initiator tRNA must be recognized as correct by IF3 and IF1, and undergoes conformational changes and positional adjustments during the various steps leading to the formation of a productive 70S*IC* from 30S*IC* (see below). Finally, although chemically equivalent to a peptidyl-tRNA, fMet-tRNA avoids hydrolysis by the scavenging enzyme peptidyl-tRNA hydrolase by virtue of its special structural features. Many of these distinctive characteristics of tRNA_fMet_ derive from particular structural elements present in its acceptor end and anticodon stem loop that were identified through a number of studies, initially carried out by pioneering selective chemical modifications (mainly by the late Dr. LaDonne H. Schulman) and later by mutagenesis primarily by the laboratory of Dr. U. L. Rajbhandary (reviewed in [65–67]).

A single synthetase (MetRS) transfers methionine to the 3′ OH of initiator tRNA and to the 3′ OH of elongator tRNA_Met_; in fact, MetRS recognizes the anticodon CAU of its substrate that is identical in initiator and elongator tRNA molecules [68–70]. However, as described below, other properties of the anticodon loops of tRNA_fMet_ and tRNA_Met_ are clearly different. The presence of three consecutive GC base pairs in the anticodon stem distinguishes the initiator tRNA from elongator tRNA. This crucial feature is highly conserved, being found in all initiator tRNAs in all kingdoms of life. These base pairs confer a particular rigidity on the anticodon stem that influences the structure of the anticodon loop of initiator tRNA and is responsible for its high affinity for the ribosomal P‐site. Indeed, two pairs (i.e., 29:41 and 30:40) of the anticodon arm make contacts with the universally conserved nucleotides G1338 and A1339 of 16S rRNA that line one side of the P-site, contributing to formation of a gate that separates the P- from the E-site together with A790, located on the opposite side [71, 72].

Equally important are the unique structural characteristics of the acceptor end of initiator tRNA_fMet_ that ensure both recognition of Met-tRNA_fMet_ by the transformylase (MTF) and resistance of fMet-tRNA_fMet_ to peptidyl-tRNA hydrolase activity [10, 68, 73–75]. In an elegant experiment, it has been shown that a chimeric tRNA constituted by the acceptor stem of initiator tRNA_fMet_ and anticodon stem loop of elongator tRNA_Met_ is fully capable of being formylated, indicating that the determinants for recognition by MTF are clustered in the acceptor stem [68, 74]. A primary determinant of the acceptor stem of tRNA_fmet_ is the absence of base pairing between G1 and A72, while a secondary determinant is represented by the A11:U24 pair in the dihydrouridine (D) stem [76]. Upon recognition of these structural elements, MTF binds to its Met-tRNA_fMet_ substrate through an induced fit mechanism [77], and causes conformational changes in three regions of the tRNA, one being the distant anticodon loop [76]. Formylation of Met-tRNA_fMet_ by MTF is important for translation initiation insofar as it represents a positive signal for the specific recognition by IF2 and a negative signal that prevents the binding by EF-Tu. Nevertheless, MTF is not an essential protein because the cells can survive in its absence, albeit at a severely reduced (ca. tenfold) growth rate and despite a ts phenotype (failure to grow at 42 °C) [78].

Early crystallographic [79] and NMR spectroscopy [80] studies indicated that the overall architecture of initiator tRNA is very similar to that of bulk tRNAs with the classical L-shape geometry and the usual tertiary interactions. However, some major differences were detected in the fold of the anticodon loop and in the position of U33; it was shown that the anticodon loop of bacterial, yeast and mammalian initiator tRNAs is cleaved by nuclease S1 at two positions (i.e., after C34 and A35), unlike elongator tRNAs that were generally cleaved at four positions (i.e., after U33, C34, A35 and U36) [81].

**Fig. 3:**
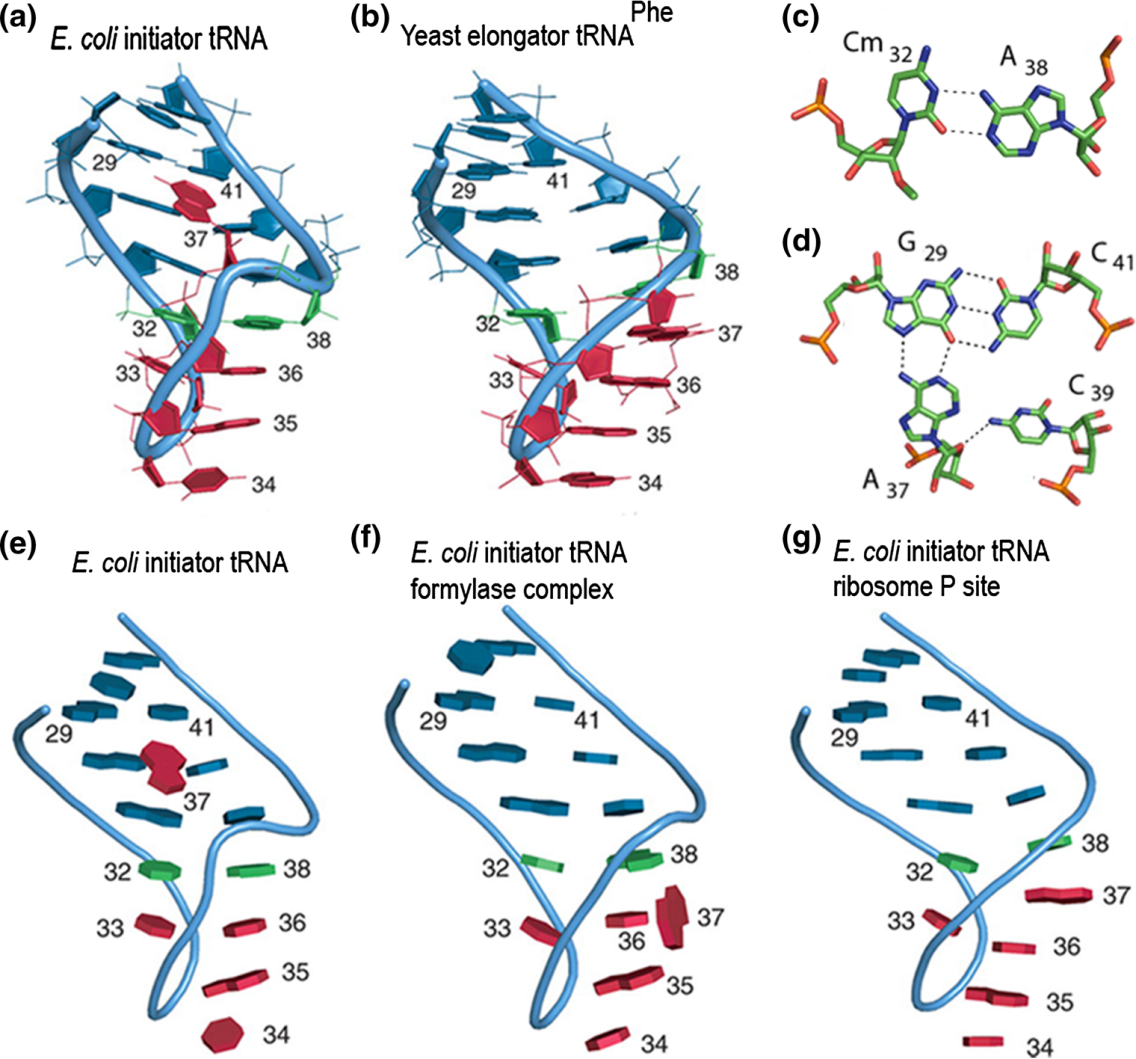
Unique characteristics of the initiator tRNA_fMet_ anticodon stem loop (ASL). Comparison between the ASL of **a** *E. coli* initiator tRNA_fMet_ and **b** elongator tRNA_Phe_. The ASL of initiator tRNA_fMet_ contains **c** a peculiar Cm32·A38 wobble base pair and **d** the A37·G29·C41 base triple. The anticodon bases undergo different stacking interactions when the tRNA is **e** free, **f** transformylase-bound or **g** P-site-bound (reproduced from [82] with permission from Oxford University Press)

More recent crystallographic studies have demonstrated that the structure of the anticodon stem loop of tRNA_fMet_ indeed adopts a non-conventional conformation, characterized by three specific features (Fig. 3a, e) not seen in elongator tRNA (Fig. 3b): (a) a triple pairing involving a base of the anticodon loop (A37) in the G29–C41 base pair of the stem (Fig. 3c) that causes a large turn in the phosphate backbone immediately after the anticodon; (b) unusual base stacking within the anticodon loop where A38, instead of being stacked on base 37 as in all other tRNAs is stacked onto U36 and c) an unusual, wobble-like Cm32-A38 base pair (Fig. 3d) stabilized by stacking onto the G31–C39 pair that extends the anticodon stem [82]. However, upon interaction with the transformylase (Fig. 3f) and with the ribosome (Fig. 3g), many of these structural characteristics, such as the aforementioned triple base pairing, are lost. In particular, in a *T. thermophilus* [71] and in an *E. coli* [72] 70S complex, the anticodon loop of tRNA_fMet_ adopts a canonical conformation, with A37 stacked between U36 and A38 when paired with the initiation codon in the P-site (Fig. 3g). Thus, the conformation of the anticodon loop is different in free and P-site-bound tRNA_fMet_ with base 37 being “unstacked” and “stacked”, respectively. It seems, therefore, likely that fMet-tRNA_fMet_ might switch between the two conformations during subsequent steps of the initiation pathway. In this connection, it has been hypothesized that a ‘37-unstacked’ conformation could be required for fMet-tRNA accommodation in the ribosomal P-site and for “passing” inspection by IF3 (see below). The stabilization of the ‘37-stacked’ conformation could be subsequently required for the correct pairing with the AUG initiation codon.

## The initiation factors: structure and structure–function relationships

### IF1

**Fig. 4:**
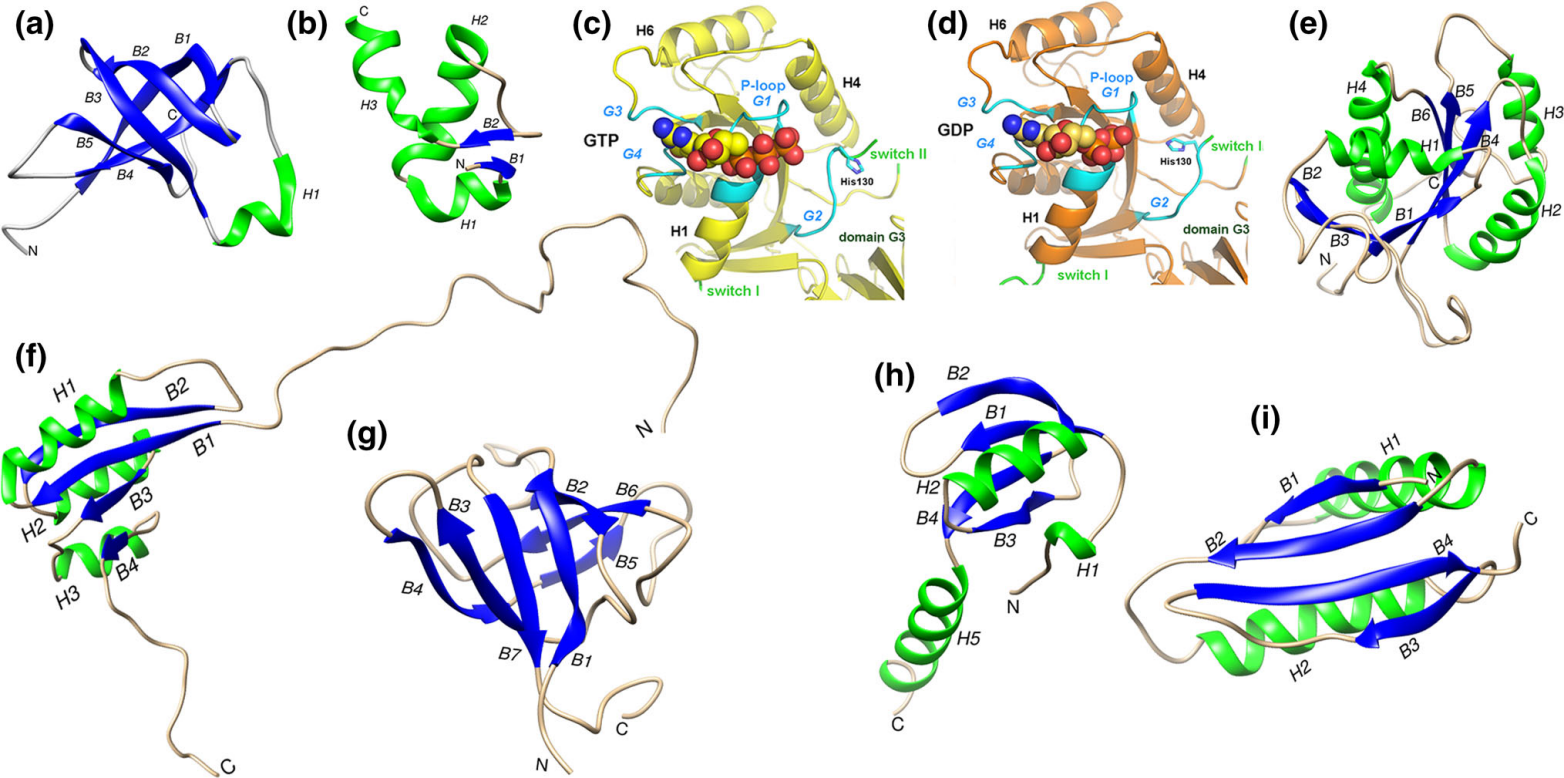
Structures of the initiation factors IF1, IF2 IF3. Structures of: **a** *E. coli* IF1 as determined by NMR spectroscopy (PDB 1AH9) [83]; **b** the N-terminal 2–50 residues of *E. coli* IF2 as determined by NMR spectroscopy (PDB 1ND9) [103]; crystallographic structures [107] of *T. thermophilus* **c** IF2-G2·GTP and **d** IF2-G2·GDP. The guanine nucleotides binding elements P-loop/G1, G2, G3 and G4 (*cyan*), switch I and switch II (*green*) are indicated; residue His130, α-helices H1, H4 and H6 as well as the position of domain G3 are also indicated (reproduced from [107]; **e** structure of the apo form of *G. stearothermophilus* IF2-G2 as determined by NMR spectroscopy (PDB 2LKC) [93]); **f** structure of *G. stearothermophilus* IF2-C1 as determined by NMR spectroscopy (PDB 1Z9B) [105]; **g** structure of *G. stearothermophilus* IF2-C2 as determined by NMR spectroscopy (PDB 2LKC) [106]; crystallographic structure of *E. coli* IF3; **h** N-terminal domain (PDB 1TIF) [113] and **i** C-terminal domain (PDB 1TIG) [113]. The N-terminus and C-terminus of the structures are indicated with N and C, respectively, the α-helices and the β-strands are shown in *green* and *blue*, respectively, and indicated with *H* and *B* letters followed by numbers, as appropriate. With the exception of **c** and **d**, molecular images were generated from PDB data using the UCSF Chimera package (http://www.cgl.ucsf.edu/chimera developed by the Resource for Biocomputing, Visualization, and Informatics at the University of California, San Francisco (supported by NIGMS P41-GM103311)

The structure of *E. coli* IF1, the smallest (71 residues) of the three initiation factors, was solved at high resolution by multidimensional NMR spectroscopy [83]; the solution structure was later confirmed within a complex with *T. thermophilus* 30S subunits analyzed by X-ray crystallography [84]. The structure of IF1 (Fig. 4a) is that of a typical OB-fold nucleic acid-binding protein and consists of a five-stranded β-barrel with a highly flexible loop connecting strands 3 and 4 and of a short 3_10_ helix [83, 84]. Chemical probing in situ of 16S rRNA [85] and X-ray crystallography [84] showed that IF1 binds in the A site of the 30S subunit, where it contacts ribosomal protein S12. Upon IF1 binding, bases A1492 and A1493 of 16S rRNA helix 44 flip out and long-range (i.e., ca. 70 Å) conformational changes of this helix takes place. Nucleotides C1411 and C1412 move laterally by 5 Å with respect to the helical axis. The C-terminus of the factor (Arg69), shown by site-directed mutagenesis to be essential in *E. coli* for the interaction with the 30S subunit [86] contacts the rRNA and Arg64 establishes electrostatic interactions that result in the disruption of base pairing, in particular of the non-canonical pairing of A1413 and G1487 [84] whose reactivity towards chemical modification increases [87]. Overall, IF1 binding produces a generalized conformational change of the 30S subunit that affects the exposure to chemical reagents of distant bases such as A908 and A909 [87] and causes head, shoulder and platform of the subunit to move with respect to each other [84].

### IF2

**Fig. 5:**
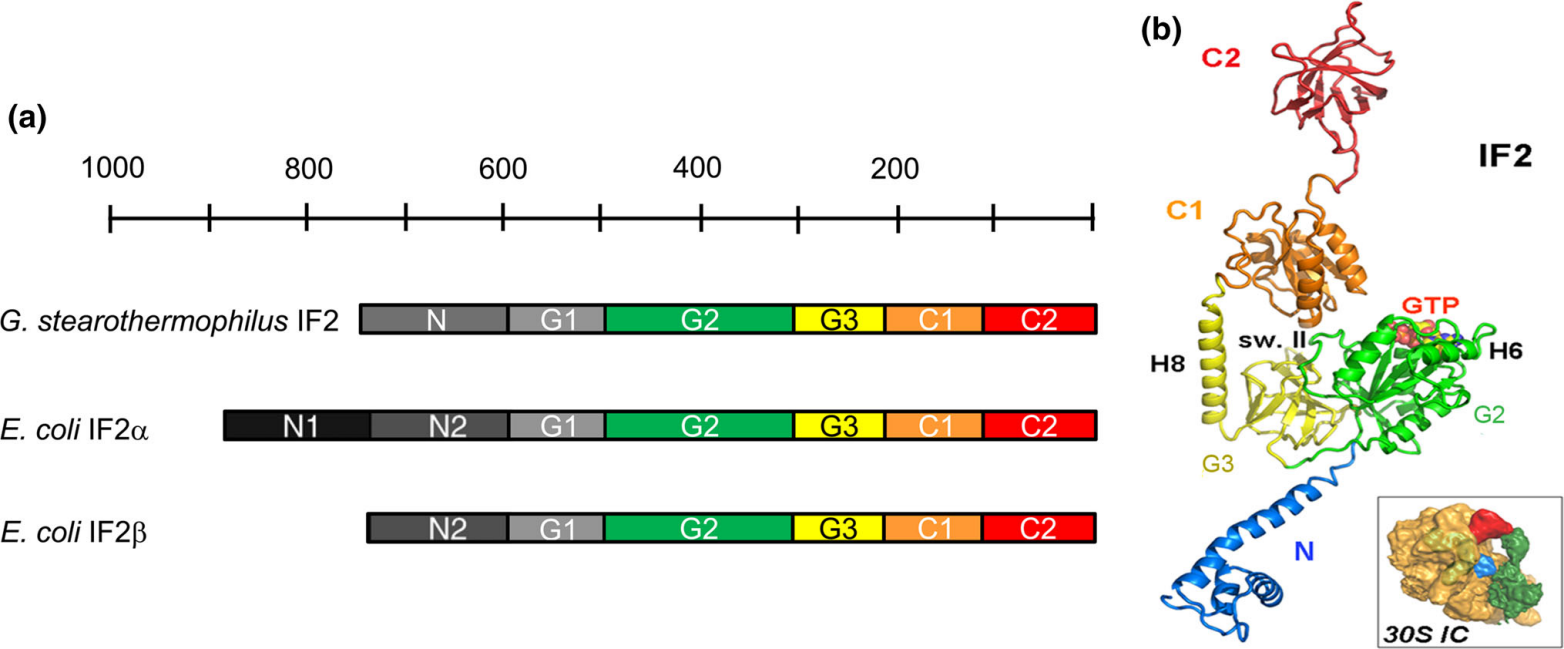
Domain composition, structure and ribosomal localization in 30S*IC* of translation initiation factor IF2. **a** Scheme illustrating the structural/functional domains constituting *G. stearothermophilus* IF2, and *E. coli* IF2α and *E. coli* IF2β. Domains G1 (*light gray*), G2 (*green*), G3 (*yellow*), C1 (*orange*) and C2 (*red*) are fairly conserved, whereas size and sequence of the N-terminal part of the molecule are not conserved although the N-terminal domain of both *G. stearothermophilus* and *E. coli* shares the property of anchoring IF2 to the ribosome [91, 92]. The number of residues constituting the IF2 molecules can be deduced from the *bar* above the scheme. **b** Overall architecture of IF2 as derived from the available crystal structure [96, 107, 108] of *T. thermophilus* IF2 (N through C1) and NMR structure [106] of *G. stearothermophilus* C2. The color code for G2, G3, C1 and C2 is the same as in **a**. The N-domain (*blue*) of *T. thermophilus* IF2 does not correspond to that of either *G. stearothermophilus* or *E. coli* IF2, but corresponds in part to N-terminal and G1 domains as described in the text. Localization of G2-bound GTP and of important structural elements such as helices H6 and H8 and switch II are indicated. Insert: localization of IF2 (*green*) in the 30S*IC* (lacking IF3). The 30S subunit, fMet-tRNA and IF1 are indicated in *ochre*, *red*, and *blue*, respectively. Reproduced with permission from [96]

The characterization of IF2 structure began with limited proteolysis experiments that revealed that this factor is a multidomain protein consisting of three major parts, an N-terminal region, a central “G region” (~40 kDa) and a C-terminal part (~25 kDa) [88]. Subsequent analyses revealed that each region is constituted by distinct domains (or sub-domains), each endowed with distinct structural and functional properties (Fig. 5a) [89].

The N-terminal region, of variable size and sequence, proved to be dispensable for all basic translational functions of IF2, both in vitro and in vivo [90] but was shown to strongly anchor the factor to the 30S ribosomal subunit that allows binding also in the absence of IF1, GTP and fMet-tRNA [91, 92].

The highly conserved central “G” region consists of three domains (G1, G2 and G3). Domain G2 is able to bind to the 50S subunit, albeit with low affinity, and contains all the structural elements responsible for binding guanine nucleotides and GTP hydrolysis [89, 93]. On the other hand, no autonomous activity could be detected for isolated domains G1 and G3. However, site-directed mutagenesis [92] and cryoEM reconstitutions [94, 95] have implicated G3 in binding to the 30S subunit, whereas the consequences of the deletion of the *T. thermophilus* N-terminal region that corresponds to a large extent to *E. coli* and *B. stearothermophilus* G1 suggest that this domain stabilizes the interaction of IF2 with the L7/L12 stalk and favors the formation of a productive 70S*IC* [96].

The C-terminal region is constituted by two domains (C1 and C2) [97]. Although no specific function could be attributed to C1, it seems likely that this domain plays an important role in communicating to the C2 domain structural changes occurring in the G2 domain (see below). Finally, C2 was shown to contain all the determinants for the recognition and binding of fMet-tRNA; the interaction was shown to involve just the acceptor end of the tRNA and to be as strong as that established by the native factor [98, 99].

For several years, the crystallographic structure of *Methanobacterium thermoautotrophicum* aIF5B, the archaeal homolog of bacterial IF2, as well as the conformational changes occurring during the transition of this factor from the apo to the GTP and from this to the GDP form [100] have been taken as a paradigm to interpret structural and functional data concerning IF2 for which structural data were lacking. Although the 3D structure of aIF5B proved useful to interpret the electron density of IF2 in cryo-EM reconstructions and in the construction of an IF2 homology model [94, 95, 101, 102], the assumption that IF2 and aIF5B may use the same molecular dynamics to perform their functions generated a number of unrealistic mechanistic models. In fact, it appeared evident that aside from their overall structural similarity the different biological properties of the two molecules must be supported by the structural differences existing between them. Following elucidation of the crystal structure of aIF5B [100], several NMR spectroscopy and crystallographic studies have been devoted to the elucidation of the 3D structures of IF2 and of its isolated domains and important differences between IF2 and aIF5B have been detected. So far the solution structures of *E. coli* N-domain [103, 104], of *Geobacillus stearothermophilus* (formerly *Bacillus stearothermophilus*) G2 [93], C1 [105] C2 [106] and G3 (R. Dongre, G. Folkers, C. O. Gualerzi, R. Boelens and H. Wienk, manuscript in preparation) have been elucidated at high resolution by NMR spectroscopy. More recently, also the crystal structures of the first 363 residues [107] and of full length [96, 108] *T. thermophilus* IF2 have been determined. However, because the C2 domain is not visible in the latter structure, a complete atomic structure of this factor is not yet available.

Comparison of the primary sequences of IF2 and aIF5B reveals that the latter molecule (594 residues) lacks both N-terminal and G1 domains and begins at a position corresponding to the N-terminus of bacterial G2 domain; furthermore, it contains some additional segments within domain G2 (e.g., between switch I and the G2 box, and between S5 and H6, in addition to having a longer H6) and in the C-terminus where two short α-helices ensure an interaction with aIF1a [109] that has no corresponding equivalent in bacterial IF2 [110].

Structural biology data indicate that IF2 is an elongated molecule, less compact in solution than in the crystals and that the characteristic chalice-resembling architecture of the four domains of aIF5B (G,II,III and IV, corresponding to bacterial G2, G3, C1 and C2) is not observed in bacterial IF2, whose domains in solution are instead arranged like beads on a string [108]. Differences in size were noticed between the apo (82 × 95 Å) and GDP (65 × 88 Å) forms of IF2 and 30S-bound IF2 is larger than free IF2 in the crystal [96, 108]. Also the analysis of *T. thermophilus* IF2 by SAXS revealed an elongated structure (maximum length 130 ± 10 Å) with a central bulky core constituted by G2/G3 and by two protrusions corresponding to domains N/G1 and C pointing in opposite directions. Furthermore, important clues as to the functionally relevant structural dynamics of IF2 were obtained from comparison of the structures of isolated and ribosome-bound IF2 in a combined approach of crystallography, cryoEM, SAXS and kinetic analyses [96].

A brief description of the structure of the individual domains of IF2 is given here below highlighting, whenever appropriate, the differences existing between bacterial IF2 and archaeal aIF5B.

#### N-domain

The structure of the first N-terminal 157 residues of *E. coli* IF2 was investigated by NMR spectroscopy. Residues 2–50 were shown to form a subdomain containing three short β-strands and three α-helices, folded to form a βααββα motif with the three helices packed on the same side of a small twisted β-sheet (Fig. 4b). In many bacteria, including *E. coli*, a second copy of this subdomain is found just before the G1 domain. Residues 51–97 present at the C-terminal side of this compact structure do not appear to form a regular structure, whereas residues 98–157 form a helix containing a repetitive sequence of mostly hydrophilic amino acids. ^15^N relaxation rates indicate that, unlike the first 50 residues that form a well ordered and compact subdomain, the other regions of this domain are significantly more mobile; the N-terminal domain tumbles in a manner that is independent of the other domains of the factor, at least in solution [99, 100].

The N-terminal part of *T. thermophilus* IF2 that corresponds to a large extent to the G1 domain of *E. coli* and *G. stearothermophilus* is composed of a 50 Å long helix (helix 3) on which two small helices are folded back [96, 107, 108].

#### G2 domain

This domain consists of an eight-stranded β-sheet flanked by six α-helices and a 3_10_ helix (Fig. 4c, d) and is structurally homologous to guanine nucleotide-binding domains of other translational GTPases such as EF-Tu, EF-G, LepA, and RF3 [93, 107, 108]. It contains the four conserved sequence elements characteristic of these proteins, namely the G1/P loop and G2, G3 and G4 loops (Fig. 4d), the latter two forming the walls of a hydrophobic pocket that accommodates the guanine moiety of GTP or GDP [93]. The P-loop and its vicinity are disordered in the apo G2 domain and is filled with H_2_O molecules but undergoes a strong conformational change upon guanine nucleotide binding. The binding entails the insertion of these molecules between the P-loop (G1 motif) itself and the G2, G3 and G4 motifs, the latter two interacting with the guanosine moiety (Fig. 4c, d). The ribose is H-bonded via H_2_O molecules to Lys218 and the terminal phosphates (β and γ of GTP, α and β of GDP) interact with switch I and switch II and with a Mg^2+^ ion whose position is the same in the IF2-G2·GTP and IF2-G2·GDP, although the β phosphate is rotated in one complex with respect to the other [93, 107, 108].

**Fig. 6:**
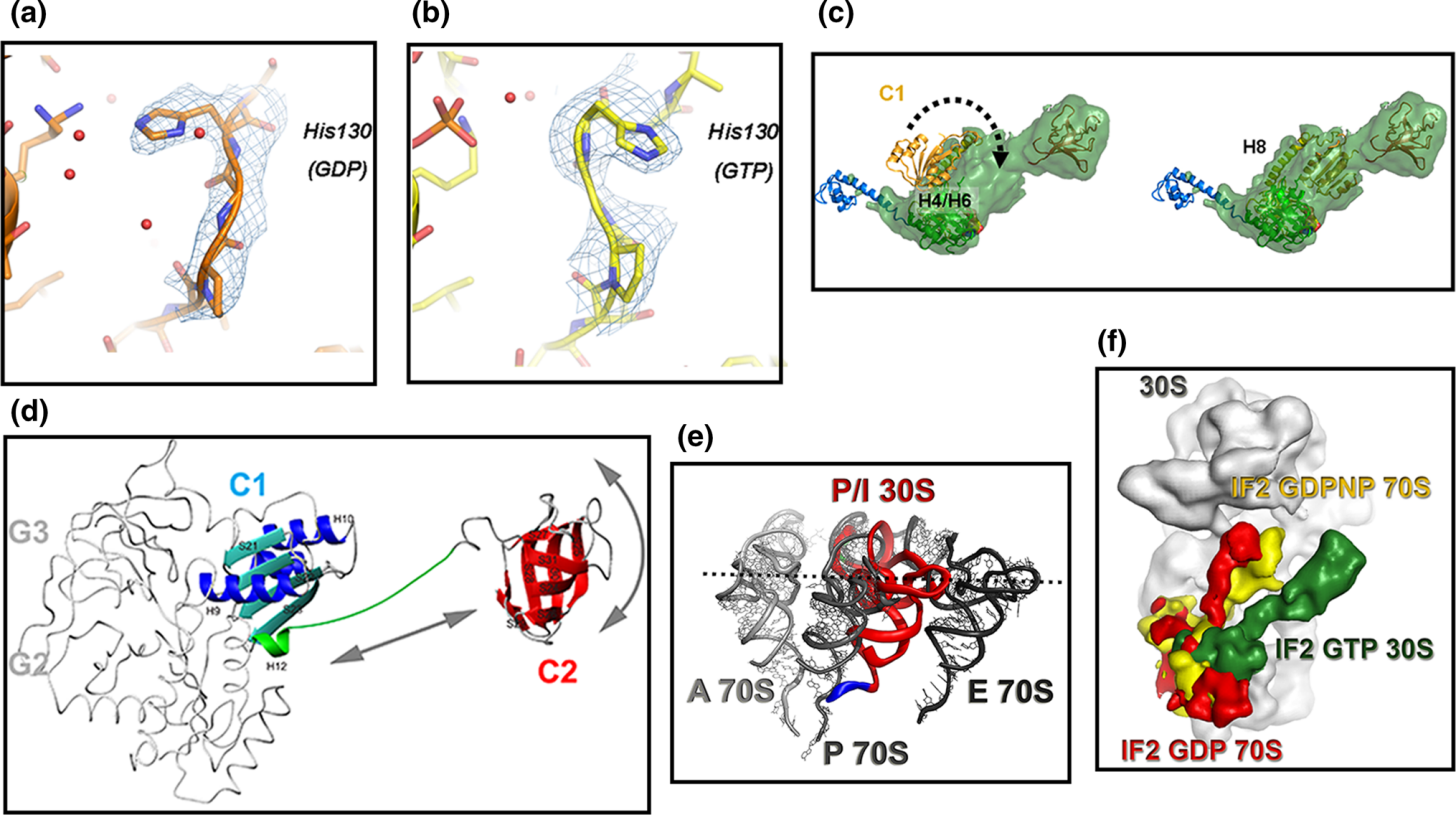
Conformational changes involving select regions of IF2 and positional adjustments of IF2 and fMet-tRNA during the assembly of a 70S initiation complex. Positions occupied by *T. thermophilus* His130 (corresponding to His448 in *E. coli* and His301 in *G. stearothermophilus*) in the IF2-G2 domain carrying: **a** GDP or **b** GTP [107]. This conserved residue, located immediately after the G2-box, is the first N-terminal residue of switch II and is implicated in GTP hydrolysis like its equivalent His80 of EF-Tu [123]. **c** A 180° rotation (*dotted arrow*) around helix H8, occurring upon binding of IF2 to the 30S subunit, changes the orientation of IF2-C1 domain on the ribosome and brings this domain close to switch II [96]; **d** backbone representation of *G. stearothermophilus* IF2-C1 (*cyan* and *blue*) and IF2-C2 (*red*) joined by the flexible connector (H12) (*green*) showing the positional mobility of the two domains with the *arrows* indicating the motional freedom of IF2-C2 with respect to IF2-C1 [93]; **e** P/I position occupied by fMet-tRNA (*red*, but for the acceptor end shown in *blue*) in the 30S*IC* with respect to the positions of A-site (*light gray*), E-site (*black*) tRNA and to the final P-site position attained in initiation dipeptide-productive 70S*IC* (*dark gray*) as deduced from cryoEM reconstitutions [95]. **f** Positions occupied by IF2 on the ribosome at different stages of the translation initiation pathway; IF2·GTP in the 30S*IC* (*green*), IF2·GDPNP in the 70S*IC* (*yellow*) and “ready to leave” IF2·GDP in the 70S*IC* (*red*) as deduced from cryoEM reconstitutions [95]. Reproduced with permission from [107] (**a**, **b**, **e**, **f**); [96] (**c**); [93] (**d**)

The ligand-dependent conformational change involves Lys86, a P-loop residue that interacts with His130 of switch II when GDP but not when GTP is bound. In IF2·GTP, a H_2_O molecule is positioned next to the γ phosphate and the conformation of switch II changes with His130 being flipped out (Fig. 6a, b). Furthermore, also the side chain of Val82 (equivalent to *E. coli* V400G whose mutation increases the affinity for GTP) moves 2.9 Å towards helix H4 that undergoes significant reorientation by rotating 7° outwards to avoid steric clash with Gln160 [93, 107, 108]. Switch I and switch II are disordered (hence not visible) when GDP is bound but assume an ordered α-helical structure when the γ-phosphate of GTP is bound.

#### G3 domain

This domain is a typical OB-fold β-barrel structural module, similar to domain IF2-C2 and to domains II of EF-Tu and EF-G. As described below, there is structural interplay between this domain and both G2 [92] and C1 [93, 96, 108] domains, the latter being connected to this domain by a long flexible linker (H8) whose propensity to form an α-helix is favored by the nature of residues 330–366 [108]. As mentioned above, isolated G3 from *G. stearothermophilus* has been studied by high-resolution NMR spectroscopy (R. Dongre, G. Folkers, C. O. Gualerzi, R. Boelens and H. Wienk, manuscript in preparation) and its structure found to be essentially the same as the crystallographic structure.

#### C1 domain

In addition to primary sequence conservation, also the overall structural organization of this domain is similar to that of domain III of aIF5B whose structure was regarded to represent a novel fold [100]. The solution structure of the core of IF2-C1 [105] is characterized by a flattened fold with a centrally located β-sheet constituted by four parallel β-strands flanked by two α-helices on one side (H11 and H10) and by another α-helix on the other (H9) (Fig. 4f). Thus, C1 contains only three α-helices instead of four like its archaeal homolog; furthermore, the S22 and S23 loops of C1 are shorter. The C-terminal portion of C1, corresponding to approximately half of the long and rigid α-helix H12 found in archaeal aIF5B, and likewise the N-terminal portion of this domain appear mobile and unstructured in solution whereas the core fold of this domain is rigid and lacks internal dynamics [105].

Aside from their overall similarities, some differences between bacterial C1 and archaeal domain III appear to be functionally relevant insofar as they indicate that the mechanism by which a conformational change occurring in the G domain is transmitted to the C-terminal region of aIF5B [100] is unlikely to occur in bacterial IF2. Thus, the amphipathic H12 α-helix of archaeal aIF5B is folded back and its hydrophobic side contacts the hydrophobic surface of the central β-sheet of C1 and this interaction confers upon the archaeal structure the rigidity necessary to support the pendulum motion that would cause domain IV to swing upon GTP hydrolysis in the G domain [100]. On the other hand, due to the differences in the primary and secondary structures of H12, no such interaction occurs in bacterial IF2; furthermore, H12 is not a continuous α-helix so that in IF2 C1 and C2 are free to tumble and orient themselves independently of one another (Fig. 6d) [93].

In addition to H12, also the linker (H8) connecting C1 and G2/G3 is believed to be involved in transmitting the conformational change from the G to the C-terminal domain in aIF5B. However, in bacterial IF2 this linker is longer, unstructured and subject to fast internal motions, at least in solution [105], whereas it is rigid in aIF5B [100]. This difference is likely due to the fact that in bacterial IF2 the two loops supporting this linker have different lengths, H9/S21 being longer and H10/S22 shorter compared to the archaeal factor [105, 108]. In the crystal structure of *T. thermophilus* IF2, there is no contact between switch II and C1, unlike with domains II and III of aIF5B although the position of C1 changes upon ribosomal binding of IF2, as described below. In the cryoEM reconstructions of the 30S*IC* [95, 96], IF2-C1 is shifted towards IF2-C2 and away from G2 and G3 and H8 is kinked (near S12 and IF1) in the same position where H8 is bent, in the position of a proline (Pro355), in the crystal structure [108].

Finally, some considerations should be made concerning the position of C1 with respect to the proximal and distal regions of the factor, namely G2/G3 and C2, respectively. In fact, various cryoEM reconstructions, NMR spectroscopy and X-ray crystallography data [93–96, 101, 111] have placed this domain in different positions (a gallery of images can be found in Fig. 2 of Ref. [108]). These differences do not seem to depend on the nature of the ribosomal complex or of the IF2-bound ligand but likely reflect an intrinsic flexibility of the H8 linker that allows C1 to occupy different positions; the fact that this domain can be found either far away from G2/G3 and close to C2 or vice versa might be relevant insofar as it may indicate that a possible retraction of C1 (and C2) towards the G2/G3 might be a movement that favors the dissociation of the IF2-C2· fMet-tRNA interaction. Of particular interest, in this connection, is the comparison of the crystal structure of IF2 with the recently refined 30S*IC* cryoEM reconstitution [96] which reveals the occurrence of a big conformational change of C1 upon the ribosomal binding of IF2. A rotation around H8 causes C1 to be flipped by 180° (Fig. 6c) so that it contacts G2 near the guanine nucleotide-binding pocket and near switch II instead of contacting H4 and H6 on the other side of G2, as seen in the crystal structure. Ultimately, C1 approaches S12 and IF1 and the contact between the end of H8 and S12 was suggested to represent a stabilizing element favoring the proper positioning of C1 and C2 to allow better IF2–fMet-tRNA interaction on the ribosome [96].

#### C2 domain

The structure of this domain, elucidated by NMR spectroscopy [106], is similar to that IF2-G3 and of domain IV of aIF5B, but for the lack of the two terminal α-helices. It consists of six antiparallel β-strands arranged to form a typical β-barrel protein (Fig. 4g). This domain is highly flexible as evidenced by ^15^N relaxation measurements and by the fact that both N-terminal and C-terminal ends as well as the β1–β2 and β4–β5 loops in whose vicinity fMet-tRNA is bound are disordered. A characteristic property of this domain that clearly distinguishes the mechanism by which IF2 and EF-Tu bind aminoacyl-tRNA is the capacity of isolated C2 to recognize specifically and bind, with increasing affinity, formyl-Methionine, fMet-AMP, fMet-ACC-5′ and fMet-ACCAAC-5′ [98, 99, 112]. Finally, genetic and NMR spectroscopy data [99] indicate that fMet is bound in a pocket formed by the conserved residues R654, Q655, F657, G667 and E713.

The complete structure of *T. thermophilus* IF2 with the indication of its various domains is shown in Fig. 5b.

### IF3

Initiation factor IF3 (180 residues in *E. coli*) is a basic protein constituted by an N-terminal (IF3 NTD) and a C-terminal (IF3 CTD) domain. The two domains are of similar size and their structures have been determined by X-ray crystallography (*G. stearothermophilus*) [113] and NMR spectroscopy (*E. coli*) [114, 115]. The structure of IF3 NTD consists of a globular α/β fold constituted by a four-stranded β-sheet onto which an α-helix (H1) is packed (Fig. 4h). The structure of IF3 CTD is similar to that of many RNA-binding proteins and is made up by a two-layered α/β sandwich fold with a βαβαββ topology with two parallel α-helices packed against a four-stranded β-sheet (Fig. 4i).

The two domains are separated by a 45 Å-long, hydrophilic, lysine-rich flexible linker. NMR spectroscopy, neutron scattering, mutagenesis and accessibility to proteolysis indicate that IF3 NTD and IF3 CTD have no contact with one another and move independently, leading to the conclusion that the linker is extended and flexible, even when IF3 is 30S-bound and that the long α-helix seen in the crystallographic structure could be due to a crystallization- and/or a temperature-induced artefact [116, 117].

## Conformational changes of IF2, role of GTP and GTP hydrolysis

Several lines of evidence indicate that the biological functions of IF2 depend upon a number of allosteric communications between its domains. Like a typical G protein, IF2 can bind one guanine nucleotide molecule (GTP or GDP or the alarmone ppGpp) [88, 118–120] and a large body of evidence indicates that the factor assumes different conformations depending on the nature of its ligand [93, 107, 108, 119]. Some of the differences between IF2·GTP and IF2·GDP have been described above and are shown in Figs. 4c, d, 6a–c.

In archael eukaryal a/eIF5B, the mechanism communicating the conformational change occurring in the G domain after GTP hydrolysis to the C-terminal region entails the swinging of a rigid lever constituted by a long α-helix (H12) connecting domains III and IV (i.e., C1 and C2) as a result of rotation of domains II and III (G3 and C1) with respect to the G domain (G2) [100]. To imagine that the same mechanism could exist also in IF2 appeared unrealistic from the beginning. In fact, a protein with the structure and rigidity of aIF5B can hardly be accommodated on the 30S subunit and docking the 50S subunit to the 30S*IC* would be impossible without profound structural rearrangements of the factor [94, 95, 101]. Decisive evidence for the inconsistency of the “pendulum swinging model” in the case of IF2 came from the comparison of the dynamics of free and linker-connected C1 and C2 that clearly showed that these domains display uncorrelated tumbling and that at least in solution there is no interaction between them (Fig. 6d) [93]. The linker connecting C1 and C2 is five residues shorter than in aIF5B and is only partially α-helical, the continuity of the helix being interrupted by a conserved Gly residue (Gly468 in *T. thermophilus*) [93, 96, 107, 108]. Furthermore, NMR spectroscopy, crystallography, SAX analyses and cryoEM data agree in indicating that the structure of bacterial IF2, both isolated and in ribosomal complexes, differs significantly from that of the crystal structure of aIF5B, its domains (G2, G3, C1 and C2) having a different organization compared to that of aIF5B [96, 100, 108]. Thus, the mechanism of inter-domain communication in IF2 and aIF5B is different, even though some of the actors involved might well be similar.

When IF2 is ribosome bound, the C1 domain is rotated 180° with respect to the position occupied in solution and is moved towards the G2/G3 domains (Fig. 6d) [96]. It is, therefore, possible that the conformational change of the G2/G3 domains is communicated to C1 through a direct contact between these domains. In turn, C1 may communicate to C2 the change, not directly, in light of the aforementioned characteristics of the C1–C2 linker, but through a conformational change of the ribosome. That IF2 may induce a conformational change of the ribosome was suggested by results reported a long time ago [121].

Compared to the GDP and ppGpp, the affinity of IF2 for GTP is lower, especially at low temperature [88, 118, 119], but this is fully compensated in vivo by the much higher concentration of this ligand compared to the other two. Therefore, it can be surmised that under favorable metabolic conditions, it is IF2·GTP that binds to the 30S subunit [118]. Furthermore, IF2·GTP has a higher (~6-fold) affinity for this subunit compared to apo IF2, IF2·GDP and IF2·ppGpp [88]. Because GTP binds to domain G2 and IF2 interacts with the 30S mainly via G3, the effect of the ligand on the ribosomal affinity of IF2 is a likely consequence of conformational cross-talking between these two domains [92]. This difference in affinity for the small subunit is not very large for native IF2 containing the NTD that obscures the “functional” interaction involving domains G2/G3 by anchoring the factor to the ribosomal subunit in a rather non-specific way [91, 92]. However, in the absence of the NTD, the difference is quite dramatic; in this case, 30S binding strongly depends upon the presence of GTP, IF1 and fMet-tRNA and only the presence of the latter two ligands allows IF2·GDP or IF2·ppGpp to bind to the 30S subunit [91, 92]. Thus, after GTP hydrolysis triggered by the association of the 30S*IC* with the 50S subunit, the G2/G3 region of IF2 acquires the GDP conformation and presumably the affinity of the factor for the 30S moiety of the 70S*IC* is considerably weakened. Indeed, in the absence of other ligands, the affinity of IF2·GDP for the 70S monomers is reduced by more than one order of magnitude compared to that of IF2·GTP for the 30S subunit [88]. A further weakening of the ribosomal affinity of IF2 is probably caused by the dissociation of IF3 and IF1 that also occurs during the 30S*IC* → 70S*IC* transition (see below). However, the strong interaction between the C2 domain and the acceptor end of fMet-tRNA prevents both the dissociation of IF2 and the adjustment of the fMet-tRNA CCA end in the P-site of the PT center. Therefore, a final conformational change and the repositioning of IF2 and fMet-tRNA on the ribosome are needed to attain the 70S*IC* configuration productive in initiation dipeptide formation (see also below). This last step is inhibited by the γ-phosphate of guanine nucleotide of non-hydrolyzable GTP analogs such as GDPNP and GDPCP and in some IF2 mutants defective in GTP hydrolysis [122, 123]. In fact, substitution of H448 and H301 in the G2 domain of *E. coli* [123] and *G. stearothermophilus* [99] IF2, respectively, abolished the GTPase activity of IF2 and conferred a dominant lethal phenotype to the cells. Both spontaneous and induced mutations suppressing the dominant lethal phenotype were isolated and mapped in the C2 domain [99] and found to diminish approximately tenfold the affinity of IF2 for fMet-tRNA. This finding is compatible with the premise that GTP hydrolysis is important to allow the dissociation of the IF2–fMet-tRNA interaction. Accordingly, cryoEM reconstructions of 70S*IC* show that IF2 assumes a “ready-to-leave” position in the presence of GDP but not in the presence of GDPNP (Fig. 6f) [94]. On the other hand, both apo and GDP forms of IF2 can efficiently perform this last step and eventually yield an initiation dipeptide at a rate similar to that determined in the presence of IF2·GTP [124]. In conflict with these conclusions, it has been reported that the kinetics of initiation dipeptide formation is drastically reduced when IF2·GTP is replaced by IF2·GDP in the reaction [125]. However, it seems likely that such a dramatic rate reduction was due to the fact that an N-terminally degraded IF2 was used in these experiments. In fact, in these studies, IF2 was purified using a procedure [126] that is known to generate IF2 molecules lacking the entire NTD, especially if the *ompT* gene has not been inactivated in the bacterial strain used to overproduce IF2 to inactivate the outer membrane OmpT protease [127]. Indeed, the rate of initiation dipeptide formation in the presence of ∆NTD IF2·GDP is approximately two orders of magnitude slower than in the presence of IF2·GTP (unpublished observation in our laboratory).

To investigate the role of GTP hydrolysis in the late stages of translation initiation, two equivalent GTPase null mutants of IF2 were generated, one in *E. coli* and another in *G. stearothermophilus.* Initiation dipeptide formation was found to occur at the same rate in the presence of wt IF2·GTP and of these IF2 GTPase null mutants [92, 128]; furthermore, a GTPase null mutant was shown to support *E. coli* growth at almost wt rate while displaying in vitro a reduced efficiency in performing the IF2-dependent steps occurring before GTP hydrolysis but not in those that normally occur after GTP hydrolysis [128]. Overall, whereas it is clear that IF2-bound GTP must be hydrolyzed and the γ-Pi dissociated to allow the dissociation of the IF2–fMet-tRNA interaction, these findings demonstrate that the energy generated by GTP hydrolysis is not necessary to drive the conformational change of IF2 that enables the factor to acquire the “ready to leave” conformation required to free the acceptor end of initiator tRNA, to clear the way for binding of the EF-Tu·GTP·aminoacyl–tRNA complex and ultimately to allow initiation dipeptide formation [124].

Both crystallographic and NMR data [93, 96, 107, 108] indicate that one of the main differences between IF2·GTP and IF2·GDP is the conformation of switch I and switch II; the γ-phosphate of bound GTP induces switch II to become less flexible and to undergo a coil → helix transition that causes His130 (His448 in *E. coli* and His301 in *G. stearothermophilus*) to flip out, away from the G2 core (Fig. 6a, b) whereas the opposite helix → coil transition, that presumably increases the entropy of the system, occurs when GDP is IF2-bound after GTP hydrolysis. The conformational change of switch II induced by GTP is probably responsible for the selective GTP-induced protection of the switch vis-à-vis trypsin digestion [129].

Isothermal titration calorimetry [119] indicated that upon GDP and GTP binding the surface area of IF2 accessible to the solvent is drastically decreased with an estimated reduction ranging from 725 to 1074 Å^2^, this effect being larger with GTP than for GDP. The difference roughly corresponds to 18–27 amino acids and the differential surface area change caused by GTP and GDP is consistent with the ordering of switch I and switch II upon binding of the first but not of the second ligand, in full agreement with the structural data. The thermodynamic parameters determined in these titrations suggest that the transition of IF2 from the GTP to the GDP conformation should be thermodynamically favored [119]. Furthermore, the thermally driven spontaneous movements of the ribosomal subunits [130, 131] may well contribute to the adjustment of both fMet-tRNA and IF2 in their final conformational state and ribosomal positioning (Fig. 6e, f), provided that the γ-phosphate, responsible for the rigid “GTP conformation” of switch II, is released [124, 132].

If the energy of GTP hydrolysis is not necessary for the IF2 functions, what could be the reason for the evolutionary conservation of the guanine nucleotide binding of the factor? Two adaptive advantages can be envisaged: (1) the intrinsic capacity of the G2 domain, in conjunction with the G3, to act as a molecular hinge represents a useful mechanical device for a factor that must adjust its structure during the 30S*IC* → 70S*IC* transition and (2) G2/G3 act as a switch that regulates translation initiation depending on the metabolic state of the cell as a function of the nature of its ligand: GTP or ppGpp [118].

## Formation of the 30S and 70S initiation complexes

Until the mid-1980s, the prevailing opinion was that the thermodynamic parameters governing the interaction of ribosomes with mRNA and initiator tRNA were key elements governing translation initiation and that the main function of the initiation factors was to modify these parameters. Accordingly, IF3 was credited with the property of physically binding natural mRNAs (as opposed to synthetic templates) to the ribosome, favoring SD–anti SD base pairing and possibly discriminating between different mRNA classes, whereas IF2 was thought to “carry” the initiator tRNA to the ribosome (e.g., see [133]).

However, several lines of evidence subsequently contributed to shape the premise, now widely accepted, that a major role in determining the mechanistics and controlling efficiency and fidelity of the multistep process collectively referred to as “translation initiation” is played by the kinetics of the interactions between the various ribosomal ligands and that the initiation factors are the kinetic effectors of the process.

Early kinetic analyses demonstrated that mRNA and fMet-tRNA bind to the 30S subunit in stochastic, as opposed to an obligatory order and indicated that formation of a 30S*IC* amenable for association with the 50S subunit to yield a 70S*IC* is preceded by the formation of a complex, defined “30S pre-initiation complex” or 30S pre-*IC*, in which both ligands are 30S-bound but not yet interacting [134]. The actual existence of an intermediate precursor complex having these predicted characteristics was subsequently documented in the case of the 30S complex with *rpsO* (i.e., S15) mRNA [135, 136], and, more recently, of the complex made in the presence of the antibiotic GE81112 [137] that was found to block the transition 30S pre-*IC* → 30S*IC* (manuscript in preparation). An additional case in which the translation initiation pathway is blocked at the level of this transition is when a complex is formed at low temperature with non-cold-shock *cspD*mRNA [138]. Formation of the 30S pre-*IC* and of the 30S*IC* can be monitored by fluorescence stopped-flow and by quenched-flow rapid-filtration analyses, respectively [139]. These two methods yield essentially the same *k*_on_ (5 µM^−1^ s^−1^) but quite different *k*_off_ (1.5 and <0.05 s^−1^, respectively), indicating that unlike the 30S pre-*IC*, that can be readily dissociated, the 30S*IC* is more stable, representing a “locked” conformation of the former complex [139]. The simultaneous presence of mRNA and fMet-tRNA in the 30S pre-*IC* can be deduced from the FRET signal between these two ligands, whereas the lack of proper codon and anticodon pairing can be deduced from their in situ accessibility to hydroxyl radical cleavage.

The transition from the 30S pre-*IC* to a locked 30S*IC* entails a first order, temperature-dependent isomerization of the 30S pre-*IC* accompanied by full P-site decoding of the mRNA initiation triplet by the fMet-tRNA. The locking mechanism likely relies on the conformational dynamics of the 30S subunit with the equilibrium “unlocked” ⇆ “locked” conformer being shifted in the presence of the IFs in either direction, depending upon the nature of the ligands (mRNA and tRNA). Indeed, locking with non-canonical ligands is kinetically antagonized by IF3 and IF1 [23]. On the other hand, regardless of the nature of the ligands, IF2 favors the locking but displays a strong preference for aminoacyl-tRNAs having a blocked α-NH_2_ group. It has been shown that to increase the IF2-initiator tRNA contacts in the 30S*IC* the acceptor end of fMet-tRNA is kinked at position C72–C73 and the terminal 3′ A is shifted ~15 Å away from the position that it would occupy in the PT center (Fig. 6e) [95]. These conformational changes of fMet-tRNA, induced by both IF2 and mRNA, could stabilize the interaction between ligands and contribute to the locking process. Locking is the rate-limiting step in 30S*IC* formation and, as mentioned above, is under kinetic control of the IFs and represents the first kinetic checkpoint of translation initiation fidelity. In fact, IF3 increases both on- and off rates of this transition to different extents depending upon the canonical or non-canonical nature/structure of the 30S ligands [140, 141]. Aminoacyl-tRNAs other than fMet-tRNA and mRNA with initiation codons other than AUG, GUG or UUG or having a too extended SD sequence are discriminated against [11, 23, 140–144]. Although IF3 is dissociated from the ribosome only during the subsequent 30S*IC* → 70S*IC* transition, its affinity for the 30S*IC* is reduced in the presence of canonical ligands [23, 58], a condition that facilitates the subsequent docking of the 50S subunit. On the other hand, in the presence of non-canonical ligands, IF3 remains more tightly bound to the 30S thereby interfering with formation of a proper and stable 70S*IC*; the subunit association step represents the second kinetic checkpoint of translation initiation fidelity [23, 141, 144].

The fidelity function of IF3 is supported directly by the aforementioned IF1-induced conformational changes of the 30S subunit [23, 84] and, indirectly, by the activity of IF2. Indeed, the latter factor contributes to fidelity by drastically increasing the on-rate of P-site binding of a fully charged aminoacyl-tRNA having a blocked αNH_2_ group, fMet-tRNA being the only cellular tRNA having this feature [143].

The mechanism of fMet-tRNA recruitment by the 30S has been the subject of kinetic analyses. Fast kinetics data proved incompatible with the model in which IF2 carries fMet-tRNA to the ribosome and indicate instead that N-AcPhetRNA (as an fMet-tRNA Ersatz) [145] or genuine fMet-tRNA [139] is recruited by ribosome-bound IF2. Thus, the mechanism by which IF2 operates is unlike that of elongation factor EF-Tu and eukaryotic/archaeal initiation factor e/aIF2 that are aminoacyl-tRNA and initiator tRNA carriers, respectively.

However, in conflict with the above conclusions, a recent fluorescent single molecule study [146] indicates that both recruitment by 30S-bound IF2 and IF2-mediated transport are possible routes of initiator tRNA binding. It seems possible that heterogeneity of the molecular components used in these experiments may have generated this situation because more or less extended degradations of the N-terminal region of IF2 produce molecules capable of binding to the 30S only in the presence of fMet-tRNA, IF1 and mRNA [91, 92].

The sequence of steps leading to *E. coli* 30S pre-*IC* formation has been elucidated in a recent study in which the kinetic parameters of all the various macromolecular interactions have been determined by fast kinetic analyses [147]. The data are compatible with a favored assembly pathway in which IF3 and IF2 are the first factors to bind to the 30S subunit, forming an unstable 30S–IF2–IF3 complex. The subsequent binding of IF1 locks the factors in a kinetically more stable 30S pre-*IC* to which fMet-tRNA_fMet_ is recruited. The transition 30S pre-*IC* → 30S*IC* is also accompanied by a substantial stabilization (~3-fold increase) of 30S–mRNA interaction entirely attributable to the establishment of complete codon–anticodon pairing in the P-site as determined by measuring the rupture force between mRNA and various ribosomal initiation complexes [148]. These experiments also confirm the premise (see above) that the thermodynamic stability of the 30S–mRNA interaction is not affected by the initiation factors (IF2 in this particular case) whereas the kinetic data confirm that, depending on its concentration and the structural determinants of its TIR, binding of mRNA to the 30S subunit is IFs independent and can take place at any time during 30S pre-*IC* assembly [62, 147].

**Fig. 7:**
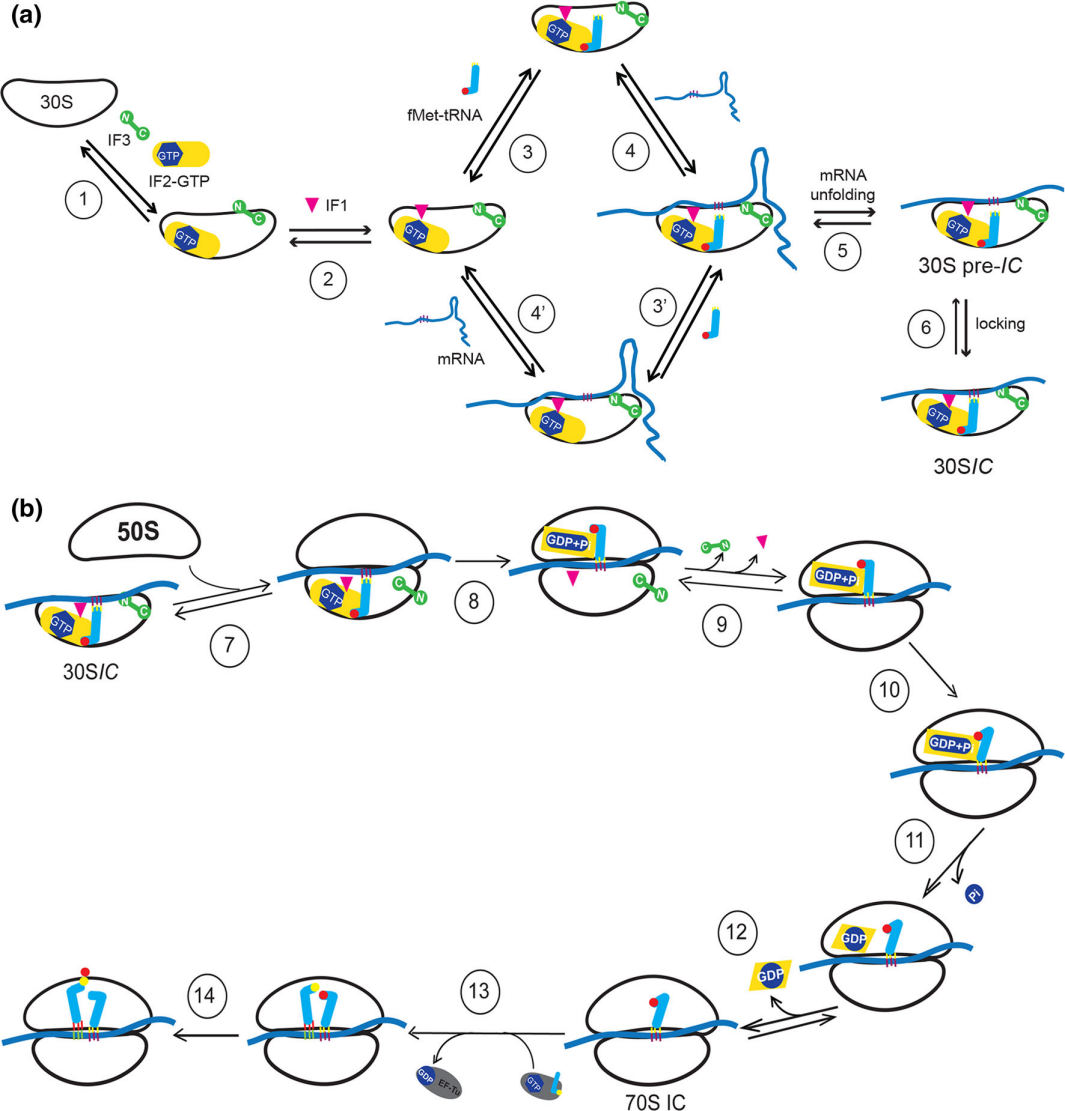
Scheme of the pathway leading to 30S*IC* and 70S*IC* formation. **a** 30S*IC* formation. Step 1: a vacant 30S ribosomal subunit binds IF3 and IF2. In both cases the binding is biphasic. In the case of IF3, a very rapid step (1000 μM^−1^ s^−1^) is followed by a fast first-order rearrangement (34–55 s^−1^). The off rates of the first and second step, regardless of the presence of IF2, are approximately 35 and 0.8 s^−1^ [147]. This biphasic IF3 binding mechanism may reflect the fact that the two domains of IF3 bind sequentially to the 30S subunit, the binding IF3CTD occurring before binding of IF3NTD [159, 160]. IF2 binding to a 30S subunit already carrying IF3 occurs with *k*_on_ = 220–320 μM^−1^ s^−1^ followed by a rearrangement (2–6 s^−1^). The off rates in the presence of IF3 alone are ~12  and ~1 s^−1^, respectively [147]. Step 2: IF1 binds in a single event with on and off rates in the presence of both IF3 and IF2 of 10–12 μM^−1^ s^−1^ and 0.02 s^−1^, respectively [141]. Steps 3 and 3′: in the presence of all three factors fMet-tRNA is recruited with *k*_on_ = 5 μM^−1^ s^−1^ and *k*_off_ = 1.5 s^−1^ [139]. Steps 4 and 4′: the mRNA is bound with different on and off rates depending on its TIR structure; mRNAs with strong secondary structures are bound more slowly than those having little or no secondary structure. On the other hand, the presence of an SD sequence and IFs does not influence either on or off rates that typically range from *k*_on_ = 6–158 μM^−1^ s^−1^ and *k*_off_ = 0.003–4 s^−1^ [62]. Step 5: mRNAs containing secondary structures must be unfolded in a process that is facilitated by IF2 bound to GTP or GDPNP and antagonized by IF3 [62]. Step 6: the isomerization of the 30S pre-*IC* allows P-site codon–anticodon interaction to yield a more stable 30S*IC* from which mRNA and fMet-tRNA are more stably bound. The locking step is under kinetic control of the IFs among which IF2 is mainly responsible for increasing the *k*_on_ whereas IF3 strongly increases the *k*_off_ (*k*_off_ = 0.004 s^−1^ with canonical ligands) [147] when the 30S ligands are non-canonical. **b** 70S*IC* formation. Step 7: a 30S*IC,* containing IF1, IF2·GTP, IF3 and mRNA whose initiation triplet is P-site decoded by fMet-tRNA, is docked by a 50S subunit with *k*_on_ = 34 μM^−1^ s^−1^ and *k*_off_ = 35 s^−1^ [132]; a very similar *k*_on_ = 12.2 μM^−1^ s^−1^ was reported in a previous study [149]. In this process, IF2 changes its conformation [94, 95, 132] and the stepwise dissociation of IF3 [160] begins. Step 8: upon contact with the GAC and SRL of the 50S subunit, the GTPase function of IF2 is activated and GTP is rapidly (k = 35–44 s^−1^) hydrolyzed leaving GDP+Pi bound to IF2 [124, 132]; as the inter-subunit bridges are progressively formed [161], the IF3NTD loses its contacts with the ribosome [160] reducing the overall ribosomal affinity of the factor by ~2 orders of magnitude [166]. Step 9: this reversible conformational transition (*k*_on_ = 24 s^−1^, *k*_off_ = 2.1 s^−1^) [132] represents the last kinetic checkpoint of translation initiation fidelity by IF3 and IF1 [23, 144] and likely coincides (at least time-wise) with the formation of the final inter-subunit bridges [132, 161]; if the ribosomal ligands are canonical IF3 and IF1 readily dissociate from the ribosome [23], IF2 undergoes a conformational change and the resulting complex is stabilized. Step 10: during this first-order isomerization (*k*_on_ = 1.5–2.3 s^−1^) that represents the rate-limiting step in 70S*IC* formation [124, 132], fMet-tRNA is adjusted on the ribosome occupying a P/I position intermediate between P/P and P/E, [94, 101, 132]. In the presence of non-hydrolyzable GTP analogs, switch II of IF2-G2 remains “frozen” in a rigid α-helical structure and the complex remains stuck in this non-productive conformation [94, 132]. Step 11: Pi is dissociated from IF2·GDP (*k*_on_ = 12 s^−1^) [132] promoting helix-coil transition in switch II and allowing IF2 to change its conformation, thereby losing its contact with the acceptor end of fMet-tRNA that is adjusted in a productive P-site position [94]. Step 12: IF2 leaves the ribosome (or moves away from the A-site) clearing the way for EF-Tu binding. Step 13: the EF-Tu·GTP·aminoacyl-tRNA complex binds to the 70S*IC* (*k*_on_ ~85 μM^−1^ s^−1^) and through a number of steps [158] (not represented here) delivers to the ribosomal A-site the aminoacyl-tRNA encoded by the second mRNA codon. Step 14: the fMet-tRNA bound in the P-site of the peptidyl transferase center of the 50S subunit donates its formyl-methionine to the A-site-bound aminoacyl-tRNA to yield the initiation dipeptide fMet-aa (*k* = 0.2–2 s^−1^) [124]

A cryoEM image of a 30S*IC* without IF3 is presented in the insert of Fig. 5b and a scheme describing the various steps that lead to the formation of a complete 30S*IC* amenable for docking by the 50S subunit is presented in Fig. 7a.

Finally, it has been noticed that in the literature the 30S*IC* is sometimes referred to as a “30S pre-initiation complex” (e.g., ref. [101]), but this definition seems to be arbitrary and incorrect on both scientific and historical accounts. In fact, while it seems trivial to remark that a 30S*IC* is indeed a precursor of a 70S*IC*, the term 30S pre-*IC* should be used only when referring to the complex having the properties corresponding to those of the 30S pre-*IC* as it was defined almost 40 years ago [134]. Aside from having a well-defined physical identity, this complex also plays a relevant role as an intermediate in the first “checkpoint” that ensures translation initiation fidelity.

The steps that mark the transition from 30S*IC* to a 70S*IC* productive in initiation dipeptide formation and the role played therein by GTP hydrolysis have been the object of several studies that have mainly used fast kinetics and, more recently, single molecule fluorescence resonance energy transfer (smFRET) analyses [122, 124, 125, 132, 141, 144, 149–154]. Furthermore, important clues concerning the events occurring upon ribosomal subunit association can be obtained from the comparison of the structural data (cryoEM and SAX) obtained with 30S*IC* and 70S*IC* [94–96, 101, 111]. Although a few disagreements exist concerning some specific aspects of the process, the overall pathway outlined in Fig. 7b seems to be the one that more closely reflects the experimental data accumulated so far.

The 30S*IC* containing its canonical ligands (i.e., fMet-tRNA, a genuine mRNA translation start site, the three IFs, and a GTP molecule bound to IF2) is very rapidly docked by the 50S ribosomal subunit (Fig. 7b, step 7) to yield an initially unstable 70S*IC* (the *k*_off_ reported are between 80 ± 10 and 34 ± 4 s^−1^) whereas the on-rates (*k*_on_) for the association step are between 34 and 12.2 μM^−1^ s^−1^ [132, 149, 150]. These rates do not depend upon GTP hydrolysis, being essentially the same in the presence of GDPNP and are only marginally affected by ionic composition and concentration [149, 150]. However, the 50S subunit associates with a complete 30S*IC* ca. ten times faster than with naked 30S, when IF3 is present both IF2 and fMet-tRNA are strictly required (>1000-fold stimulation) for fast association [149, 150]. The effect of IF2 and fMet-tRNA can be explained at least in part by a ~25 % increase of the surface available for interaction with the 50S subunit provided by these two 30S ligands [95]. However, simple geometric considerations based on available cryoEM reconstitutions indicate that both conformation and position of IF2 on the ribosome must be changed to allow 30S*IC*-50S association. Contact between IF2 and the GAC (GTPase Activating Center) and the SRL (Sarcin Ricin Loop) of the 50S subunit [94, 101, 155–157] triggers a very rapid (30–45 s^−1^) IF2-dependent GTP hydrolysis (Fig. 7b, Step 8) [124, 132, 150].

The γ-Pi produced in the IF2-dependent reaction is not released instantaneously but only after a fairly long delay (~200 ms) and during this time lag IF2 remains on the 70S ribosome with bound GDP-Pi [124, 132]. During this time, as the complex undergoes the structural modifications necessary but not sufficient to become productive in initiation dipeptide formation, several conformational and positional changes of the ribosome and of its ligands (fMet-tRNA and IFs) occur. A rapid (*k*_on_ = 10–24 s^−1^) and reversible (*k*_off_ ~2 s^−1^) isomerization of the complex (Fig. 7b, Step 9) involves both structure and position of IF2 that moves with respect to the GAC of the 50S subunit, its G1 domain being shifted by 12 Å towards the NTD of L11 (i.e., from 72 to 60 Å) [150]. Although not required, GTP hydrolysis accelerates somewhat this IF2 movement [150]. The subsequent slower (*k*_on_ = 1.5–2.3 s^−1^) first-order isomerization (Fig. 7b, Step 10) likely represents the rate-limiting step in 70S*IC* formation and entails a conformational and/or positional change of fMet-tRNA monitored by fluorescence stopped flow kinetics [124, 132, 150]. The position occupied by the initiator tRNA following this adjustment likely corresponds to that seen in the cryoEM reconstitutions of the non-productive 70S complex formed in the presence of the non-hydrolyzable GTP analog GDPNP and is intermediate between a P/P and a P/E position (Fig. 6e) [94, 101]. It is likely that at this stage a canonical 70S complex is stabilized by the ejection of IF3 and IF1 (see below) and by the IF2-dependent locking of the associated subunits resulting in a further stabilization of the ribosome–mRNA interaction [148].

In the presence of non-hydrolyzable GTP analogs, the 70S complex formed at this stage remains stuck in a non-productive conformation likely because switch II of IF2-G2 remains “frozen” in a rigid α-helical structure [94, 119, 132] so that IF2 remains bound to fMet-tRNA and the latter cannot act as a donor in peptide bond formation while EF-Tu cannot bind to the IF2-blocked A-site. As to the nature of the IF2–fMet-tRNA interaction within a 70S*IC* before GTP hydrolysis, the two available cryoEM reconstructions led to different conclusions. In fact, the C2 domain of IF2 is seen in contact with the tRNA acceptor end in one case [101], and with the D-loop in another [94]. It is possible that the presence/absence of IF1 and IF3 is responsible for these differences. The dissociation of γ-Pi from IF2·GDP (*k*_on_ = 12 s^−1^) [124, 132] promotes helix–coil transition in switch II [96, 107, 108, 119] and allows IF2 to change its conformation, thereby losing its contact with the fMet-tRNA whose acceptor end can now be accommodated in a productive P-site position (Fig. 7b, Step 11) [94] while IF2 leaves the ribosome (Fig. 7b, Step 12), or remains ribosome-bound but moves away from the A-site (B. Cooperman, personal communication), clearing the way for EF-Tu binding and delivery of aminoacyl-tRNA [124, 158] encoded by the second mRNA codon to the ribosomal A-site (Fig. 7b, Step 13). In turn, this A-site-bound aminoacyl-tRNA acts as an acceptor of formyl-methionine from the donor fMet-tRNA bound in the P-site of the peptidyl transferase center to eventually yield the initiation dipeptide fMet-aa (*k* = 0.2–2 s^−1^ depending on experimental conditions) (Fig. 7b, Step 14) [124], the rate of initiation dipeptide formation [124] being lower than the rate of transpeptidation during elongation [158].

## Dynamic aspects of initiation factors and fMet-tRNA interactions with the ribosome and translation initiation fidelity

Preliminarily, it should be remarked that the stage on which the various actors play their roles in the translation initiation pathway is not a fixed ribosomal structure but rather a dynamic object, endowed with intrinsic conformational flexibility that undergoes spontaneous, thermally driven (or ligands-induced) motions with the small subunit undergoing head rotations and the two subunits capable of ratcheting [130, 164]. Such movements accompany (or drive) various movements of the participating ribosomal ligands that undergo a number of interconnected structural and positional changes along the route leading to the formation of the initiation dipeptide.

Physical evidence for the actual occurrence of at least some of the movements of the ribosomes and ribosomal ligands that accompany the subsequent phases of 70S*IC* formation have been provided by fluorescence and light scattering changes monitored by fast kinetics analyses and by the comparison of cryoEM reconstitutions of the 30S*IC* and 70S*IC* obtained at different stages of the translation initiation pathway. For instance, during the 30S*IC* → 70S*IC* transition, the 30S subunit rotates counterclockwise by 4° in a movement similar to ratcheting [94, 101]. Also the position of fMet-tRNA on the ribosome is subject to various adjustments (Fig. 6e). All cryo-EM data agree that in the 30S subunit the anticodon loop is correctly placed in the P-site [94, 101, 111] but the anticodon stem is slightly rotated clockwise and somewhat distorted and bent towards the initiation codon and the tRNA elbow shifted towards the E-site [94]; furthermore, the acceptor end is held by IF2 away from the position in which it could act as a donor in peptide bond formation in the 70S*IC*. Thus, the fMet-tRNA position does not correspond to the classical P-site but is instead in a position referred to as P/I that is intermediate between the P/P and the P/E states [94, 101]. In the 70S complex before GTP hydrolysis, the C2 domain of IF2 precludes classical P-site binding of fMet-tRNA and whereas the anticodon loop is in the 30S P-site the acceptor end occupies the I-site in the 50S subunit. After γ-Pi release from IF2, a positional adjustment resulting in placement of fMet-tRNA into the P-site occurs; in this process, the initiator tRNA undergoes a 20° rotation about its axis through the anticodon loop that displaces the fMet moiety by 28 Å [94, 104]. A somewhat different picture emerges from another cryoEM reconstitution [111]; in fact, whereas also this study concludes that the acceptor arm and CCA end of fMet-tRNA cannot not occupy the classical P site due to their interaction with IF2-C2, their position surprisingly is found to be shifted towards the A-site instead of the E-site [111]. This finding suggests that the position of the fMet-tRNA on the 70S may not be rigidly fixed until after its final adjustment in the productive P/P site.

In situ rRNA probing with chemical reagents or cleavage with hydroxyl radicals, cryo-EM reconstitutions and X-ray crystallography have been used to identify and characterize the ribosomal binding sites of the three IFs. The results are straightforward for IF1 that, as mentioned above, was localized in the 30S A-site by chemical probing as well as by X-ray crystallography. On the other hand, to define the binding sites of IF2 and IF3 proved to be more difficult. In fact, IF3 binding induces dynamic flexibility to the 30S subunit whereas IF2 has binding sites on both subunits unlike IF1 and IF3 and changes its position depending upon the stage of the initiation pathway (Fig. 7) to participate in and promote subsequent functions.

As mentioned above, fast kinetics analyses using FRET signals as observables and cryoEM reconstructions showed that both fMet-tRNA (Fig. 6e) and IF2 (Fig. 6f) undergo positional and conformational readjustments during the 30S*IC* → 70S*IC* transition. Changes of both position and conformation of these ligands have been detected upon docking of the 50S subunit to the 30S*IC*, after the hydrolysis of GTP, after the IF2-dependent stabilization of the mRNA–ribosome interaction that presumably follows the dissociation of IF3 and IF1 and after the release of γ-Pi that allows the dissociation of the C2–fMet-tRNA interaction. These conformational/positional changes are also accompanied by conformational changes of the ribosome [94, 101].

Before GTP hydrolysis, IF2-G2 contacts h8 and h14 of 16S rRNA, near the inter-subunit bridge B8, and the 30S*IC* exposes this domain to an incoming 50S so as to favor 50S docking and trigger the GTPase activity of IF2. IF2-G3 contacts h17 and is close to h5 and h15; IF2-C1 contacts h3, h5 and h15 while IF2-C2 contacts h44, H69 and H89 and holds P-site-bound fMet-tRNA through an interaction with the tRNA acceptor end [94, 95].

Upon subunit association and after GTP hydrolysis, the 30S subunit is rotated by 5° counter-clock wise, assuming a nearly post-translocation position; IF2 also rotates along its long axis and loses several contacts with the ribosome while others are established. IF2-G2 is shifted outward by 10 Å and rotated counter-clockwise by 20°. Consequently, this domain separates from the SRL, loses its contacts with h8 and h14 and approaches protein L6. IF2-C1 loses contacts with h5 and h15. Most important, also the contacts of IF2-C2 are modified, its interactions with h44 and with fMet-tRNA being lost. In addition, the contacts with H38 and H91 are lost while those with H69 and H89 are preserved. Overall the contacts between the ribosome and IF2 are substantially reduced after GTP hydrolysis and the factor assumes a “ready to leave” position [94].

To localize precisely the IF3 binding site on the 30S ribosomal subunit proved to be a difficult task as different studies yielded somewhat conflicting conclusions [111, 160, 162, 163, 165]; the likely reason for this situation is that this factor accelerates (or induces) dynamic movements of the subunit that are likely an intrinsic property of the subunit itself [130, 164] so that it may be difficult to pin down its precise position on the 30S subunit. Furthermore, the factor is composed of two domains of almost identical size whose identification in cryoEM reconstitutions may be problematic, especially if their structures in solution or in the crystals do not correspond entirely to those assumed when the factor is ribosome bound. Thus, rather than analyzing the differences between the proposed ribosomal locations of IF3, the main uncertainty concerning primarily the position occupied by IF3NTD, it seems appropriate to note that there is general agreement that IF3 contacts the subunit at two separate sites and that IF3CTD binds to the platform, protecting very efficiently the G700 region (G700, U701, G703, A706 and C708) from chemical modification and hydroxyl radical cleavage [85, 160, 165]. Furthermore, it seems important to recall that NMR titration experiments and time-resolved 16S rRNA probing experiments indicate that the two domains bind sequentially to the 30S subunit, first IF3CTD then IF3NTD, and that dissociation of IF3 upon 30S–50S association proceeds in the reverse order of binding, namely that the NTD is the first domain to dissociate [159, 160].

Moreover, it has been shown that isolated IF3CTD can perform all the various functions of the intact factor, at least in vitro but that the lack of the NTD reduces by ~2 orders of magnitude the affinity of the factor for the 30S subunit [166, 167]. These properties of the two domains of the IF3 molecule and the ribosomal step-wise association/dissociation of the factor offer a key to understand the mechanism by which the factor functions in controlling translation initiation fidelity. In fact, it can be surmised that after IF3CTD binding to the 30S subunit, the interaction is stabilized by the subsequent binding of IF3NTD and that “fully bound” IF3 performs its function in controlling the kinetics of the 30S pre-*IC* ⇆ 30S*IC* transition. The establishment of correct (canonical) base pairing in the P-site would induce a locked structure of the complex whose conformation would partially interfere with the IF3NTD-30S interaction, thereby weakening the overall ribosomal affinity of the factor. Association of a canonical 30S*IC,* best fit for the association with the 50S subunit, would then promote the rapid sequential formation of inter-subunit bridges that would completely displace the NTD, further weaken the IF3 interaction and eventually determine the dissociation of the factor. On the other hand, in the presence of non-canonical 30S ligands, the structure of the faulty complex would interfere less with IF3NTD binding and IF3 would remain more tightly bound to the 30S complex. In this way, IF3 would have additional time and opportunity to promote dissociation of the incorrect (or incorrectly coded) aminoacyl-tRNA. This dissociation could occur through the direct influence that IF3 exercises on the 16S rRNA bases that control the opening/closing dynamics of the P/E gate, namely G1338, A1339 [168, 169] and A790 [160] whose mutations were shown to decrease translation initiation fidelity [170]. Furthermore, the presence of a more stably bound IF3 in non-canonical 30S complexes would antagonize more effectively both subunit association and 70S complex stabilization (Steps 7 and 10 of Fig. 7), thereby allowing the factor to control translation initiation fidelity also at the level of these two checkpoints [23, 144].

An interesting question concerns the molecular basis for the discrimination operated by IF3 vis-à-vis non-canonical complexes. According to a hypothesis put forward by the Gold laboratory several years ago [171], IF3 would physically inspect and recognize the peculiar properties of the anticodon stem of initiator tRNA (see above). That this might be the result of a physical contact between this structure and IF3 is rather unlikely, despite some claims for the existence of such a direct interaction [101, 111]. In fact, even if one takes for granted that a physical contact between IF3NTD and the ASL of fMet-tRNA indeed exists, this model clashes with the evidence that isolated IF3CTD (that for sure does not touch the fMet-tRNA ASL) is capable of rejecting non-canonical ligands with the same specificity as the intact factor [166, 167]. Furthermore, fidelity function based on a physical recognition of the initiator tRNA ASL cannot explain the large diversity of 30S complexes, some containing genuine fMet-tRNA, that for one reason or another are rejected as non-canonical by IF3 (reviewed in [6]). The common denominator of these complexes is likely a deviation of their structures from the canonical geometry of a best fit initiation complex. In light of these considerations and of a large amount of empirical evidence, it must be concluded that the only mechanism that can explain all functions of IF3 is a factor-induced conformational change of the 30S subunit [160, 161, 172, 173] as equilibrium perturbation experiments indicated decades ago [174]. Indeed, since then a large amount of data have shown that IF3 affects the conformation of the 30S subunit. For instance, IF3 was shown to affect three intra-subunit UV-induced 16S rRNA crosslinks in the 30S decoding region, namely C1402–C1501, C967–C1400 and U793–G1517 and to reduce 2–4 fold the crosslinks between C1400 and U34 of tRNA_fMet_ and between C1397 and nucleotides +9 and +10 of mRNA [175]. IF3 also increases the exposure of G1487 to kethoxal [85] and affects the conformation of helix 44 [172]. Finally, whereas intact IF3 protects from chemical modification and hydroxyl radical cleavage the A790 region (C783, A784, G791, U793, A794), likely through a direct contact of its NTD, its isolated CTD that is active in the fidelity function has the opposite effect and increases the exposure of the same residues (A794 > C783 > A784 > A790). This suggests that upon binding to the platform IF3CTD can induce a long distance conformational change in the P-site decoding region of the subunit [160] that is likely important for the fidelity function of the factor.

Thus, the peculiar features of the fMet-tRNA ASL would be recognized only indirectly by IF3 as a result of the greater stability conferred upon the 30S complexes that allow them to withstand successfully the effects of IF3 on the conformational dynamics of the 30S subunit. In turn, bases G1338 and A1339 of 16S rRNA would be instrumental in increasing the stability of the complexes carrying P-site-bound fMet-tRNA by selectively forming type II and type I A-minor interactions with the G–C pairs of the ASL of initiator tRNA [168, 170].

Together with IF3, also IF1 offers an important contribution to translation initiation fidelity [23, 172, 173] targeting not so much the first kinetic checkpoint of initiation fidelity (i.e., the 30S pre-*IC* ⇆ 30S*IC* transition) but instead the second checkpoint, namely the docking of the 50S subunit to the 30S*IC*. The influence that IF1 and IF3 exercise on the conformation of 30S subunit is again at the basis of the synergic action of the two factors. More precisely, the conformation of h44, of A1408 in particular, is affected by IF1 and IF3 [84, 172, 173]; it has been suggested that the two factors induce a “docking unfavorable” structure whereas canonical 30S*IC* formation shifts the conformational equilibrium towards formation of a “docking favorable” structure [23, 173]. The effect of IF1 and IF3 is counteracted by the aminoglycoside streptomycin that causes a conformational change of the opposite sign in the same region of the 30S subunit [23, 84, 173].

Whereas the timing of ribosomal binding of the three IFs has been determined in kinetic experiments [147], quantitative data concerning their dissociation are still lacking.

A 40-year-old model purporting a mutual incompatibility of IF3 and fMet-tRNA on the 30S subunit [176] has recently been reproposed [151]. However, as it was demonstrated that the claimed IF3/fMet-tRNA incompatibility was caused by an artefact due to the use of an N-terminally truncated factor [177, 178], also the more recent revival of the vintage model turned out to stem from the use of an mRNA containing a too extended SD sequence regarded as non-canonical by IF3 [23]. Instead, FRET signals between fMet-tRNA (donor) and IF3 (acceptor) used as observables in kinetic analyses demonstrated the simultaneous presence of both ligands on the same 30S subunit and clearly showed that IF3 is dissociated during the 30S*IC* → 70S*IC* transition [23]. What is true, on the other hand, is that formation of a canonical 30S*IC* decreases the affinity of IF3 for the 30S ribosomal subunit, in preparation for the dissociation that occurs upon subunit association [23, 58]. There are several indications that the ejection of IF3 is not an all or none process, but proceeds in steps that probably coincide with the progressive formation of the inter-subunit bridges [160, 161]. IF1 was shown to bind to the 30S subunits but not to the 50S or 70S ribosomes and 30S–50S association was found to promote its efficient dissociation [14, 86, 179]. These data clearly indicate that also IF1 dissociation occurs during the 30S*IC* → 70S*IC* transition likely immediately after the dissociation of IF3 whose presence increases its affinity for the 30S subunit [179]. IF2 is the last factor to abandon the ribosome and the conditions for its dissociation have been discussed above.

## Initiation at the regulatory crossroad

A number of circumstances suggest that fMet-tRNA and IF2, in addition to having a key and direct function in initiating protein biosynthesis, may also play an important role in coupling protein biosynthesis with transcription and replication as a function of the metabolic state of the cell. Indeed, the initiator tRNA, being aminoacylated with methionine that is subsequently formylated, uses two precursors that are at the core of cellular nutritional metabolism with a large number of potential regulatory implications. Methionine is involved, through its derivative SAM, in transferring –CH_3_ to RNA, DNA, proteins, lipids and during polyamine biosynthesis and the endogenous biosynthesis of methionine itself has the highest energetic cost (i.e., 7 ATP and 8 NADPH) compared to all other amino acids [180]. In *E. coli* cells growing in the absence of exogenous methionine, ~8 % of the total protein synthetic capacity is used to produce MetE, the last enzyme of the Met biosynthetic pathway [181] and the activity and cellular level of the methionine biosynthetic enzymes and the availability of methionine itself limit the overall rate of protein synthesis and cellular growth [181, 182].

On the other hand, the formyl group of fMet-tRNA derives from 10-FTHF, a key element of the folate cycle involved in one-carbon pool metabolism, essential for the synthesis of purines, dTMP, RNA, DNA and, in turn, also connected to the methionine cycle. Once formed, fMet-tRNA interacts specifically with IF2 that has the properties of a sensor of the nutritional state of the cell since its G2 domain can bind the alarmone ppGpp in alternative to GTP, resulting in translation initiation inhibition [118].

To close the circle, it should be mentioned that fMet-tRNA_fMet_ was found to alter promoter selection by *E. coli* RNA polymerase, inhibiting rRNA and stable RNA transcription and favoring instead transcription from the *lac* promoter [183], whereas IF2 was found to strongly stimulate rRNA production while having little effect on the synthesis of other RNA species [184]. Because the transcriptional inhibition by fMet-tRNA_fMet_ resembles that of ppGpp [183], it is possible to speculate that a blockage of translation initiation due to ppGpp binding to IF2 would free the stable fMet-tRNA molecules that would feedback a negative signal to the transcriptional apparatus whereas the resumption of translation initiation would engage all the available initiation factor and possibly generate an excess of free IF2 that could stimulate stable RNA transcription. Although the relevance of these old data should be confirmed by in vivo studies, it seems to make sense that such a complex, coordinate regulatory circuit indeed controls major macromolecular syntheses as a function of the metabolic state of the cell.
